# Emotional bookkeeping and differentiated affiliative relationships: Exploring the role of dynamics and speed in updating relationship quality in the EMO-model

**DOI:** 10.1371/journal.pone.0249519

**Published:** 2021-04-02

**Authors:** Tonko W. Zijlstra, Han de Vries, Elisabeth H. M. Sterck

**Affiliations:** 1 Animal Behaviour and Cognition (formerly Animal Ecology), Department Biology, Utrecht University, Utrecht, The Netherlands; 2 Ethology Research, Animal Science Department, Biomedical Primate Research Centre, Rijswijk, The Netherlands; University of Michigan, UNITED STATES

## Abstract

Emotional bookkeeping is the process by which primates integrate the emotional effects of social interactions to form internal representations of their affiliative relationships. The dynamics and speed of this process, which comprises the formation, maintenance and fading out of affiliative relationships, are not clear. Empirical data suggest that affiliative relationships are slowly formed and do not easily fade out. The EMO-model, an agent-based model designed to simulate the social life of primates capable of emotional bookkeeping, was used to explore the effects of different types of internal relationship dynamics and speeds of increase and decrease of relationship strength. In the original EMO-model the internal dynamics involves a fast built-up of a relationship independent of its current quality, alongside a relatively fast fading out of relationship quality. Here we explore the effect of this original dynamics and an alternative dynamics more in line with empirical data, in combination with different speeds of internal relationship quality increase and decrease, on the differentiation and stability of affiliative relationships. The alternative dynamics leads to more differentiated and stable affiliative relationships than the original dynamics, especially when the speed with which internal relationship quality increases is low and the speed with which it decreases is intermediate. Consequently, individuals can groom different group members with varying frequency and support a rich social life with stable preferred partners and attention to several others. In conclusion, differentiated and stable affiliative relationships are especially formed when friends are not made too quickly and not forgotten too easily.

## Introduction

Affiliative relationships play a key role in the lives of many group-living animals [1–3]. These relationships are both expressed by grooming and sitting in close proximity as well as formed and maintained through these behaviours. Affiliative relationships, also called social bonds or friendships [4,5], are found both among kin and non-kin partners [2,6–9] and can be maintained over extended periods of time [primates: 8,10–14; elephants: 15; and dolphins: 16–19]. Strong affiliative relationships provide benefits, such as better access to mating partners or food resources, more effective territory defence and a lower risk of predation [3,20–22] and increases reproductive success and longevity [22–26]. Especially in primates, the presence of affiliative relationships has been recognized as a key component of the social organisation of primate groups. Furthermore, both empirical and theoretical studies have shown that both humans and many non-human primate species live in diversified social structures, typically characterised by a smaller number of high-quality relationships and a larger number of low-quality relationships [27–31]. However, how long-lasting affiliative relationships are formed, maintained and fade out is unclear, especially regarding the role of the internal cognitive/emotional processes.

In primates, several mechanisms have been proposed that lead to the formation of affiliative relationships. In order of increasing cognitive/emotional requirements, they may result from repeated chance encounters [32], from social symmetry such as age or dominance [33], from emotional bookkeeping when the emotional impact of previous interactions guide future interactions [10,34,35], and from calculated reciprocity [33] when memories of previous social interactions lead to reciprocation. While emotional bookkeeping requires individual recognition of group members and the capacity to integrate interactions into a single emotional evaluation of that group member, it does not require episodic-like memory [36]. Empirical data suggest that in primates these requirements for emotional bookkeeping are met. Primates recognize the affiliative relationships between themselves and others [37] and relationships among other individuals [37–41]. It seems clear that animals have at least some form of internal representation of their group members. In addition, Agent Based Models (ABM), such as the EMO-model [42], indicate that the differentiated affiliative relationships (i.e. relationships in which there is a categorical difference between high quality and low quality relationships) as found in empirical studies in primates can result from emotional bookkeeping. Such differentiated affiliative relationships will be found when individuals have friendships with some but not with other group members. However, how exactly this internal bookkeeping process regulates the formation of a relationship, its maintenance and its fading out is largely unknown.

Several studies suggest that the quality of an affiliative relationship may affect the physiological state of individuals in an interaction. Indeed, Barbary macaques show increased physiological stress levels when group members with whom they have a weak affiliative relationship move into proximity [43]. Rhesus macaque heart rate responses to social interactions, that reflect stress, are lower when approached by kin than by non-kin [44]. A study on wild chimpanzees found that levels of oxytocin, a hormone linked to bond formation, are higher after grooming with a preferred partner compared to after grooming with a less preferred partner [45] and also after food sharing with a preferred partner relative to a less preferred partner [46]. Since emotional bookkeeping is based on the emotional impact of social interactions, these physiological differences related to differences in the quality of affiliative relationships may therefore affect and reflect the dynamics of emotional bookkeeping.

In addition, there are studies that suggest that the stability of affiliative relationships depends on relationship quality. In female baboons, relationships among most preferred partners are more stable than relationships among less preferred partners, which are often changing from year to year [47,48]. In male chimpanzees both the quality of an affiliative relationship and its stability are correlated with grooming symmetry [12]. However, bonobo dyads with a strong affiliative relationship show less direct short-term reciprocity yet have a more balanced relationship in the long-term than dyads with a weak social relationship [49]. This indicates that the dynamics of strong and weak affiliative relationships differ and that the emotional impact of an interaction depends on the current quality of the relationship. If strong specific relationships provide clear fitness benefits, dynamics that are more beneficial to the formation and maintenance of such relationships could evolve through natural selection. Therefore, specific dynamics may have evolved depending on which type of relationship is most valuable in terms of fitness.

**Fig 1:**
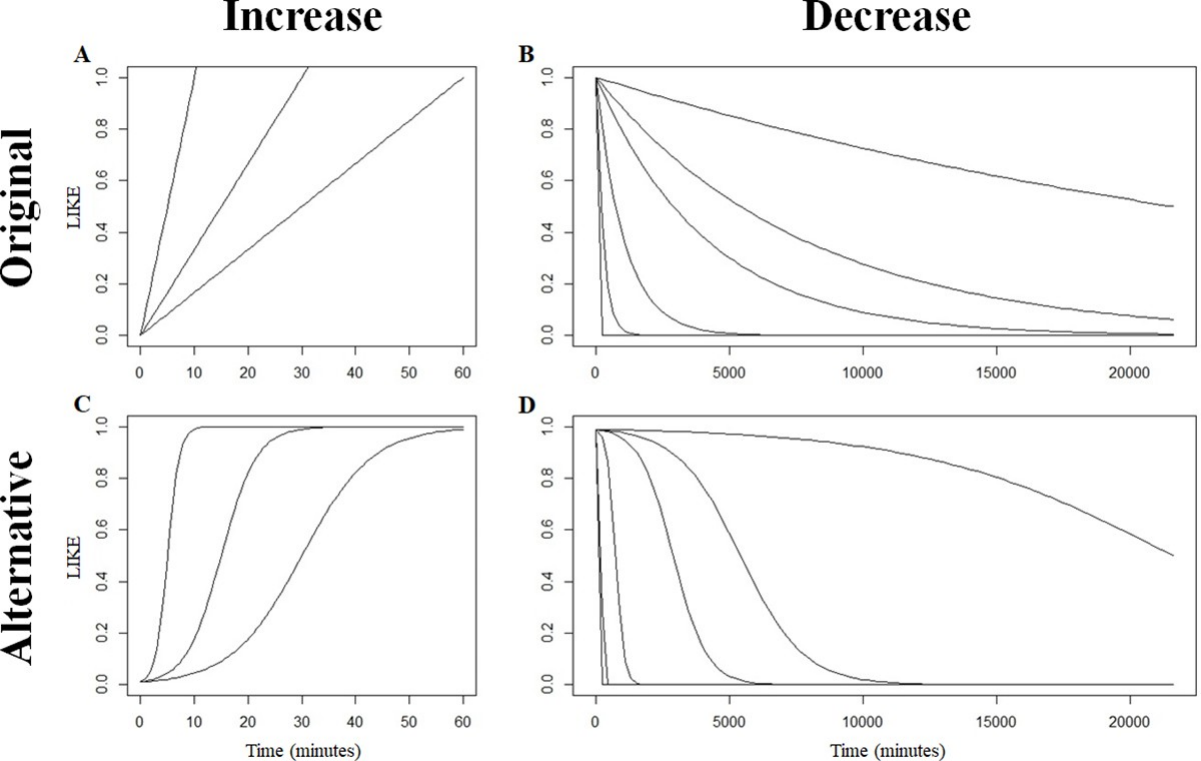
The increase and decrease of LIKE for the original and alternative dynamics for the different increase and decrease speeds. The two figures on the left show three different increase speeds, from left to right: Fast, intermediate and slow. The figures on the right show 6 different decrease speeds, from very fast on the left to very slow on the right. The increase figures assume uninterrupted grooming and the decrease figures assume that no grooming occurs.

These findings from empirical research concerning the dynamics of emotional bookkeeping can be studied well using Agent Based Models (ABMs). An ABM is a well-established tool to investigate the mechanisms that determine the structure of a complex system of interacting actors [50,51]. Because these mechanisms are explicitly formulated in an ABM, the causation of emerging patterns can be investigated extensively. An added advantage of ABMs is that they do not require harming the welfare of any experimental animals. Several studies that used ABMs found the formation of individual-specific reciprocal grooming relationships [32,36,42,52]. In the EMO-model [36,42,53], all simulated individuals are capable of emotional bookkeeping. The model individuals base their partner-specific valuation, called the ‘LIKE’ attitude, on received grooming. LIKE determines the likelihood of affiliative behaviour towards this specific partner [36]. In this model reciprocal grooming relationships emerge when grooming and LIKE within a dyad are mutually reinforced. This occurs only in a limited part of the parameter space, when the actors are very selective when choosing a partner [42] and when LIKE values faded out with intermediate speed [53]. Only in these parameter settings did actors obtain a LIKE value that was differentiated between individuals, necessary for differentiated affiliative relationships. Altogether, the dynamics of the original EMO-model [53, Fig 1A] allows affiliative relationships to build up easily, yet is not very accommodating for maintaining affiliative relationships, since they fade out quickly. In this paper, we explore how these original EMO-model dynamics affect the establishment of affiliative relationships compared to alternative dynamics.

In the EMO-model with the *original dynamics* of gaining and maintaining LIKE, the speed at which LIKE increases is independent of the quality of the affiliative relationships (Fig 1A). In addition, the original decrease dynamics of LIKE assume that strong affiliative relationships are less stable over time than weak social relationships (Fig 1B). In contrast, empirical studies suggest that relationship quality may affect the dynamics of emotional bookkeeping in the opposite direction, namely that increasing the emotional valuation requires relatively more grooming for a very weak relationship (e.g. an unfamiliar individual) and that strong affiliative relationships are more stable over time [5,43,45,46, see below]. This indicates that the original dynamics of the EMO-model differs from those suggested by empirical work. Therefore, we will explore the effect of *alternative dynamics* of formation and maintenance of LIKE that reflect empirical findings. To this end, the increase and decrease of LIKE both follow a logistic curve (Fig 1C and 1D). When the increase of LIKE during grooming follows a logistic curve, the increase will be slow for affiliative relationships with a low LIKE value. This models the potential negative influence of stress on the formation of social bonds [43]. Relationships with a high LIKE value have a faster increase speed (cf. [45,46]), since these studies found higher levels of oxytocin after interactions with preferred partners. Decrease of LIKE also follows a logistic curve. This curve is characterised by a slow decrease of LIKE for relationships with a high LIKE value and a fast decrease speed for relationships with an intermediate LIKE value. This represents higher stability of strong affiliative relationships and the fact that less preferred partners change more often over time (cf. [5]). While a range of different speeds at which LIKE can decrease has been investigated [53], the EMO-model used a single (very fast) increase speed. Because this original increase speed was chosen arbitrarily, this study also investigated two slower increase speeds.

This study investigates in an agent-based model of emotional bookkeeping (the EMO-model, [36]) how the formation and maintenance of long-lasting partner-specific affiliative relationships (in the EMO-model represented by the ‘LIKE’-attitude) is affected by the type of dynamics determining how LIKE increases and decreases and by the speed with which LIKE increases and decreases. To do this, the speed with which LIKE decreases and the level of partner selectivity were systematically varied, following Evers et al. [53]. This was done for both the type of dynamics used in the original EMO-model and the proposed alternative dynamics as well as three different increase speeds of LIKE. To answer which temporal conditions are necessary and sufficient for the formation of individualised affiliative relationships and their maintenance over an extended period of time, the following questions were investigated. First, what is the influence of dynamics and speed on the LIKE distribution and the differentiation between high and low LIKE relationships. Second, how do dynamics and speed influence the temporal stability of the LIKE distributions. Third, how do dynamics and speed influence the parameter space that results in simulations with long lasting affiliative relationships.

## Methods

The EMO-model is an agent-based model that, based on macaque behaviour, simulates agents that possess the same behavioural rules while each individual agent has its own variable internal states [36]. These behavioural rules and internal states determine their actions and interactions with other individuals in their environment. Internal states are in turn updated after social interactions. The EMO-model has rules that dictate what an individual perceives, how an individual moves relative to the other individuals in the environment and how it acts toward other individuals. Individuals are able to perform affiliative (groom, affiliative signal) and agonistic behaviour (attack, aggressive or submissive signal), besides being able to move around (leave, approach, avoid, random walk). The internal states consist of emotional states and partner specific emotional attitudes.

The three dimensions of the emotional state are arousal, anxiety and satisfaction. Arousal determines the chance that an individual will select an active behaviour instead of resting behaviour. Satisfaction increases as a result of grooming, which will simultaneously increase the partner-specific emotional valuation, or LIKE attitude. Individuals also have a fixed partner-specific FEAR attitude that is determined by the difference in dominance in a dyad, consistent with a stable dominance hierarchy. The environment of the EMO-model is purely social. The behaviour of individuals, their emotional states and LIKE attitudes are all exclusively influenced by interactions between individuals. Furthermore, LIKE attitudes are only influenced by affiliative behaviour, not by agonistic interactions. Any possible influence of activities or emotional states unrelated to social interactions (e.g. foraging, hunger) are not considered.

The EMO-model was adapted in NetLogo 5.3.1. The original model is described by Evers et al. ([36,42]: provides a full description of the model) following the ODD protocol [54]. This methods section includes a general description of the EMO-model and a detailed description of LIKE attitudes. It describes in detail how LIKE attitudes change over time and how they are affected by the original dynamics of the EMO-model (i.e. [36]) and the new alternative dynamics that was inspired by empirical data on wild chimpanzees, baboons and macaques. Lastly, the methods section will address the simulation experiments and statistical analysis.

### General description

We simulated the movements and interactions of 20 primate-like model individuals, that are characterised by several state variables. These variables concern ‘waiting time until next action’, ‘attention to others’, ‘dynamic emotional states’, ‘dynamic emotional attitudes towards others’ and ‘dominance strength’. The individuals do not vary in gender or age and kinship relationships have not been specified.

Individuals differ in state variables that change dynamically over time, such as their scheduled time until a next action (myTIME), their scanning probability (myPscan) and the width of their view angle (myVIEW_ANGLE). Furthermore, these individuals are defined by their emotional state. The emotional state consists of arousal, anxiety and satisfaction (myAROUSAL, myANXIETY and mySATISFACTION; [36]) which change dynamically due to social interactions. The changes in satisfaction are integrated into the partner-specific LIKE attitude that therefore also changes dynamically. The partner-specific FEAR attitude is fixed and determined by an individual’s dominance strength. Individuals have a fixed dominance strength ranging from 0.05 ($\frac{1}{20}$ for the lowest ranked individual) up to 1 ($\frac{1}{1}$ for the highest ranked individual) which represents a stable dominance hierarchy.

The model environment is a continuous two-dimensional grid (300 x 300 grid units). This grid has a torus shape to exclude the influence of border effects. The length of one grid unit represents 1 meter, and one time-step represents 1 minute. The model only simulates the active part of an individual’s day and therefore a day is defined as 12 hours. A year is defined as 350 days and a month as $\frac{1}{12}$^th^ of a year (approximately 29 days). Simulations were run for 504000 time-steps (2 years) after a stabilization period. Because a higher LIKE-HISTORY WEIGTH (LHW), i.e. a slower decrease speed of LIKE, requires the model to run for a longer time before LIKE attitudes stabilise, the stabilisation period prior to the 2-year data-recording period was longer for higher LHW values (i.e. at least 6000 time steps and for high LHW values: 30 * LHW * minutes).

### Increase and decrease speed of LIKE and partner selectivity

Both different speeds of increase (LINC) and decrease of LIKE (LHW cf. [53]) were studied as well as various levels of partner selectivity (LPS).

To study variation in the increase speed of LIKE, a new parameter, LIKE-INCREASE (LINC), was created. LINC varies the increase speed of LIKE. A ‘fast’ LINC corresponded with the increase speed used by Evers et al. [53] (i.e. 10 minutes of grooming is required to increase LIKE from its minimum to its maximum value). An intermediate LINC was 3 times slower than the fast LINC (i.e. it took 30 minutes of grooming) and a slow LINC was 6 times slower than the fast LINC (i.e. it took 60 minutes of grooming). We study these different speeds of increase with both the original dynamics and the alternative dynamics.

Using the original EMO-model [53], the decrease of LIKE (LHW) and LIKE-PARTNER SELECTIVITY (LPS) were varied. LHW describes the period LIKE is retained at a level above 0.5 (starting at 1). LPS describes the extent to which LIKE influences the probability of an individual choosing a specific other individual to groom, i.e. an individual’s selectivity. A higher LPS, i.e. more selectivity in partner choice, will result in an individual more often choosing to groom partners with a high LIKE value over partners with lower LIKE values. An LHW of 0 causes the LIKE to decrease very quickly (i.e. it decreases from the highest to the lowest value within 50 minutes) and with an LHW of 21600 LIKE decreases slowly (i.e. LIKE will decrease to half of its original value in 21600 MINUTES, or approximately one month). We study these different speeds of decrease with both the original dynamics and the alternative dynamics.

### LIKE and its dynamics

In this model, emotional bookkeeping is simulated by the capacity of individuals to increase and maintain LIKE. LIKE values are an individual’s emotional valuation of a specific group member. LIKE is dynamic and changes over time. The increase and decrease of LIKE were both varied in two different ways: their dynamics, i.e. the original or alternative dynamics, and their speeds.

First, this study will investigate two ‘dynamics’ of LIKE: the original dynamics (cf. [36]) and alternative dynamics (Fig 1) that represent two different ways in which LIKE may increase or decrease within the timeframe determined by LINC or LHW (compare Fig 1A–1D). The increase of LIKE follows either a linear (original dynamics) or a logistic (alternative dynamics) curve (see Table 1 for an overview of the different formulas used). If a linear curve is used, the increase speed of LIKE is constant. Conversely, the increase speed of LIKE depends on the value of LIKE itself if a logistic curve is used. Without grooming, LIKE is retained over time either following an exponential curve (original dynamics) or a logistic curve (alternative dynamics). The decrease speed of LIKE always depends on the value of LIKE itself, regardless of which dynamics is used. An exponential decrease is characterised by rapidly decreasing high LIKE values and relatively slowly decreasing low LIKE values. The crucial difference is that for a logistic curve, the highest LIKE values and the lowest LIKE values increase and decrease very slowly, while intermediate LIKE values change rapidly. While the logistic curve used in the alternative dynamics approaches 0 and 1, it never reaches either of them. To avoid that LIKE is unbounded an arbitrary lower and upper limit were chosen (i.e. 0.01 and 0.99).

**Table 1:** The different formulas that describe the increase and decrease of LIKE.

	Increase speed (LINC)	Decrease speed (LHW)
Linear	*LIKE*(*x*) = a*x	-
Exponential	-	$LIKE(x)=0.5^{\frac{x}{LHW}}$
Logistic	$LIKE(x)=\frac{1}{1+e^{-k*x}}$	$LIKE(x)=1-\frac{1}{1+e^{-k*x}}$

*x* is time in minutes, ‘k’ and ‘a’ are measures of the steepness of the curve.

The original dynamics uses a linear formula to calculate increase of LIKE and an exponential formula to calculate its decrease. The alternative dynamics uses a logistic formula to calculate both increase and decrease of LIKE. Note that the alternative formulas are inspired by empirical data. See S1 Table for the values of ‘k’ and ‘a’ that were used for the different increase and decrease speeds.

The original and alternative dynamics differ in the ease with which LIKE increases and decreases and therefore models differ in how easy affiliative relationships are formed and maintained. If the increase of LIKE follows a linear curve and decrease of LIKE follows an exponential curve (original dynamics, as used by Evers et al. [36]), then the amount of grooming necessary to form or strengthen a relationship is the same for all dyads in the group. Under these conditions a stronger relationship requires more frequent grooming to maintain. If increase and decrease of LIKE both follow a logistic curve (alternative dynamics), both the formation and the maintenance of the relationship is impacted by the quality of the relationship. Initial formation of a relationship is very slow, while it is relatively easy to improve an intermediate relationship (i.e. LIKE increases more rapidly once LIKE reaches above a certain threshold) and to maintain a high-quality relationship.

Several simulation runs were performed that combined a linear increase (original dynamics) with a logistic decrease (alternative dynamics) (dynamics 3), and a logistic increase (alternative dynamics) with an exponential decrease (original dynamics) (dynamics 4) to determine whether the changes in the increase and decrease dynamics separately had an effect on the differentiation of affiliative relationships.

A high LIKE score increases an individual’s probability of performing affiliative behaviour towards a specific other individual. This influences the frequency with which these two individuals interact, which in turn determines whether or not a high LIKE attitude is maintained. Because grooming rates and LIKE attitudes are directed from an actor to a receiver, they are by definition not symmetrical within a dyad.

### Simulation experiments

In this study, three different increase speeds (LINC), six different decrease speeds (LHW) and 4 different levels of partner selectivity (LPS) were systematically varied for two different dynamics (see Table 2 for an overview of all parameter settings used in this study). This resulted in a total of 144 simulation runs with different combinations of parameter settings. For each of these combinations, two independent simulation runs were performed. The two different simulation runs resulted in very similar distributions of LIKE, grooming and proximity. Results from the first simulation run are shown. In addition, to determine whether an observed difference between the two dynamics is due to the changes in the dynamics of increase or decrease, simulations were performed using dynamics 3 and dynamics 4 for two different increase speeds (fast & slow), an intermediate decrease speed (LHW = 2880), and a very high level of selectivity (0.99).

**Table 2:** Overview of parameter settings used in this study.

*Dynamics*	original; alternative; dynamics 3; dynamics 4
*LINC*	Fast (1/10); Intermediate (1/30); Slow (1/60)
*LHW*	0 or 25; 180; 720; 2880; 5400; 21600
*LPS*	0.5; 0.9; 0.95; 0.99

LINC: The different levels of increase speed of LIKE (in LIKE/minutes of being groomed).

LHW: The number of minutes it takes for LIKE to decrease to half its value; LHW = 0 for the original dynamics, corresponds to LHW = 25 for the alternative dynamics.

LPS: The general probability for affiliative behaviour as a function of the LIKE attitude.

### Analyses

To quantify the level of differentiation of LIKE values, we categorized the LIKE values (averaged over the ‘second year’ of the recording period) in three groups: high LIKE values (LIKE > = 0.75), intermediate LIKE values (0.25< LIKE< 0.75) and low LIKE values (LIKE = < 0.25). For each group we counted the number of relationships. Subsequently, we calculated an average LIKE value for these three groups separately. To investigate the stability of LIKE over time, LIKE distributions at 5 points in time, separated by half a year, were used (start of the recording period, half a year, one year, one and a half years and the end of the recording period). To assess stability, an R^2^ was calculated for each transition (i.e. between the LIKE distribution at the start of the recording period and the LIKE distribution after half a year, between half a year and one year, et cetera). The resulting four R^2^ values were averaged and this averaged value was subsequently compared between the original and alternative dynamics for 3 different increase speeds and LHW = 2880. Finally, the averaged R^2^ of simulation runs using 4 different levels of selectivity were compared for the alternative dynamics with a slow increase speed and intermediate decrease speed (2880). Statistical analyses were performed in R 3.2.3 [55].

### Validation

No group-level properties are implemented in this model, which only contains rules dictating individual behaviour. All group-level properties are emergent properties that are determined by the interactions of the individuals in the model. The EMO-model was validated by comparing results to empirical data on several species of free-living macaques (*Macaca spp*.; [36]).

## Results

### Distributions of LIKE

**Fig 2:**
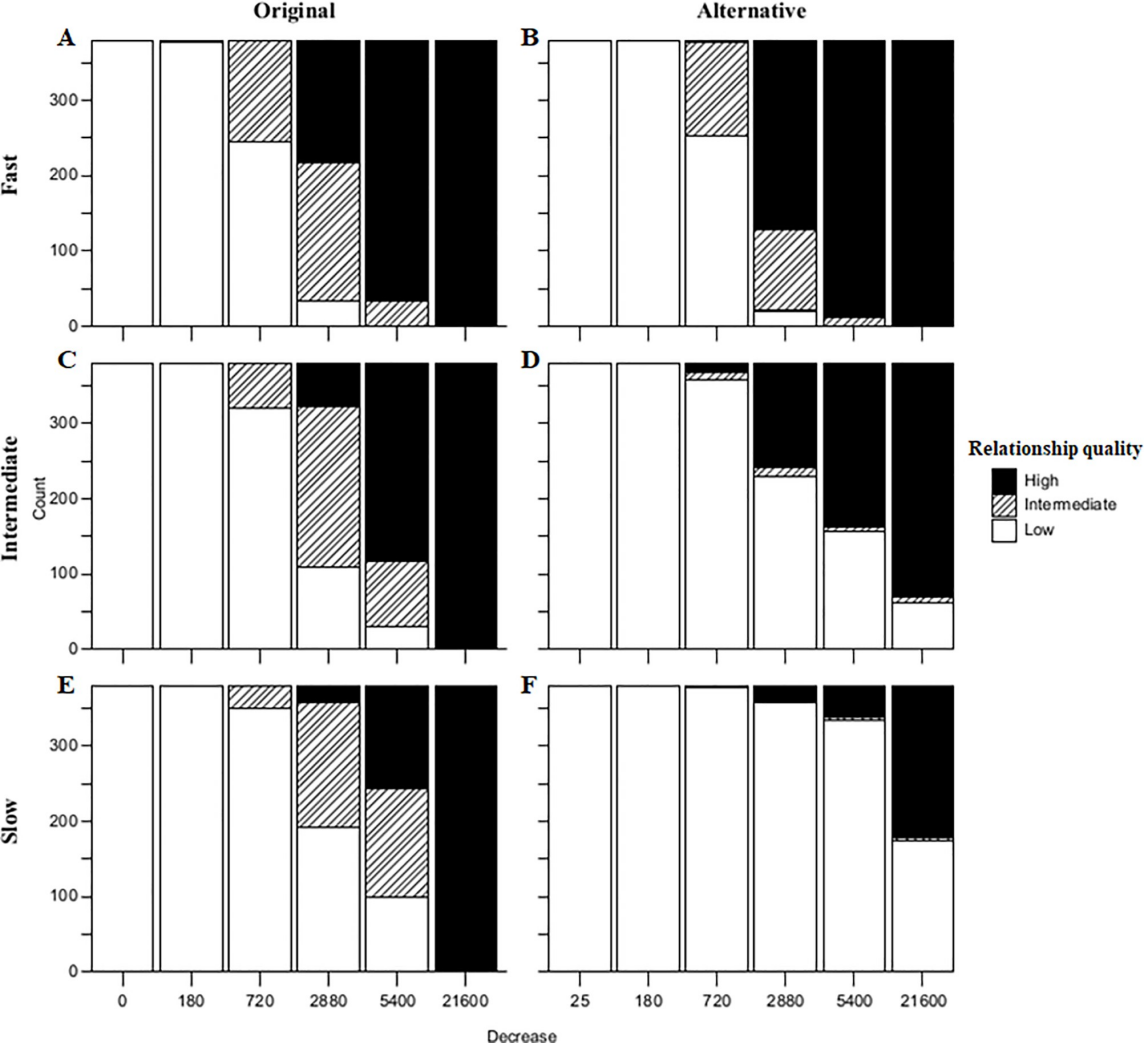
The number of relationships categorised as high, intermediate and low quality for the original and alternative dynamics for the three increase and six decrease speeds with very high partner selectivity (LPS = 0.99). Each bar represents all 380 dyadic relationships in a single simulation run.

To determine which parameter settings resulted in differentiated high-LIKE and low-LIKE relationships (i.e. strong and weak affiliative relationships), the dyadic LIKE values, the number of relationships categorised as high, intermediate and low quality, as well as the average LIKE of these groups were visually assessed. Simulation runs with the original dynamics and fast increase of LIKE replicated the outcomes found by Evers et al. [53], who used an older version of NETLogo. For these original dynamics and fast increase combined with very fast decrease speeds of LIKE (0 and 180), all dyads in the group have very low LIKE values (close to 0) (Fig 2A). These settings combined with very slow decrease speeds (5400 and 21600) result in simulation runs in which every relationship has a high LIKE value (Fig 2A). The only settings that give patterns similar to empirical observations of long-lasting partner-specific grooming are an intermediate decrease speed (720) and a very high level of selectivity (0.99) (Fig 2A). Since very slow decrease speeds, very fast decrease speed and low levels of selectivity do not result in differentiation of high-LIKE and low-LIKE relationships, the comparison between the original and alternative dynamics for the three different speeds of increase will be shown for decrease speeds 720 and 2880 (both intermediate speeds of decrease) and partner selectivity 0.99 and 0.95 (see S1 Fig for a complete overview of dyadic LIKE-values for all parameter settings).

**Fig 3:**
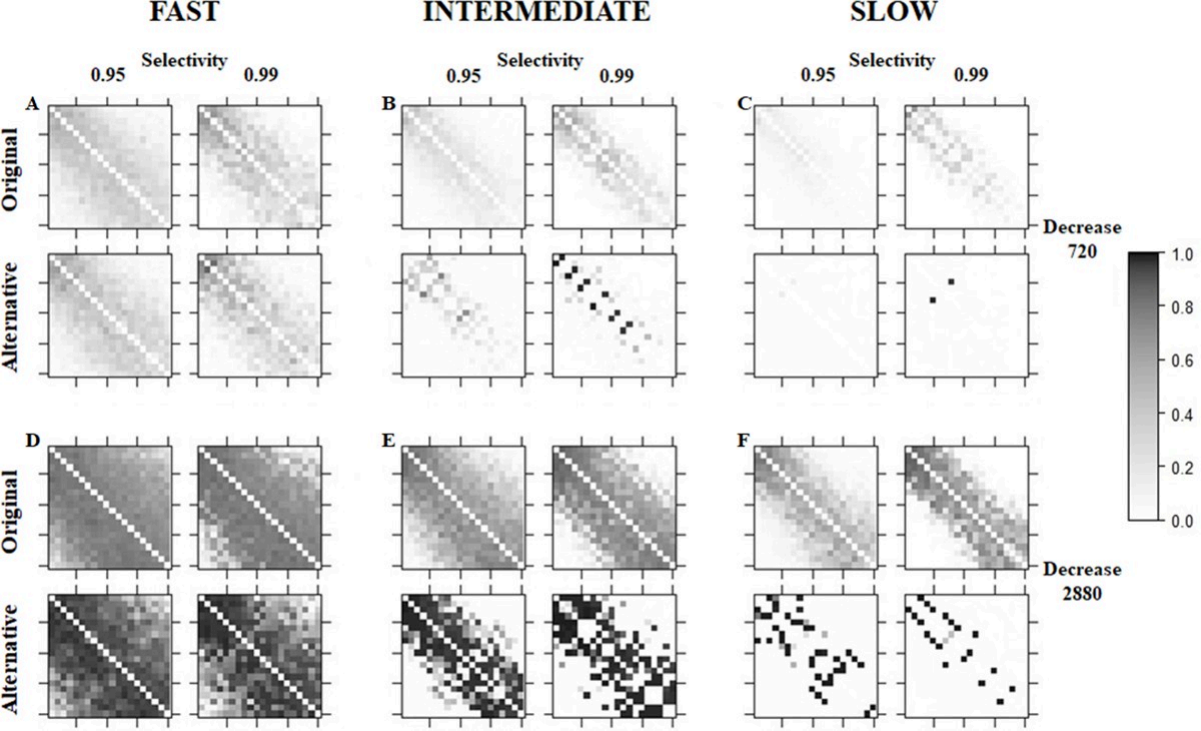
Dyadic LIKE values averaged over the “second year” of the recording period for two different levels of partner selectivity (LPS) and two different decrease speeds (LHW) compared between the original and alternative dynamics and three different increase speeds. On the y-axis individuals are ordered from low ranking (top row) to high ranking (bottom row). On the x-axis individuals are ordered from low ranking (left) to high ranking (right). Each square represents LIKE from one individual to another. LIKE ranges from 0.99 (black) to 0.01 (white).

The alternative dynamics gives, combined with a fast increase speed, a pattern in affiliative relationships that is similar to the original dynamics (Figs 2A, 2B, 3A and 3D). Still, the alternative dynamics results in relatively more differentiation between high-LIKE and low-LIKE relationships, when compared to the original dynamics (i.e. high-LIKE relationships have higher LIKE-values and low-LIKE relationships have lower LIKE values). This is illustrated by the fact that the original dynamics result in a higher number of intermediate relationships compared to the alternative dynamics, which is also true for different levels of partner selectivity (see S2 Fig). Furthermore, the difference between average LIKE values of intermediate and high quality relationships is consistently larger when the alternative dynamics are used (see S3 Fig).

The influence of a slower increase speed was assessed by comparing the three different increase speeds. The original dynamics in combination with a fast increase speed results in differentiated LIKE attitudes when the decrease speed is intermediate (LHW = 720). However, when the original dynamics was combined with an intermediate or slow increase speed and a slower intermediate decrease speed (LHW = 2880) this resulted in a more differentiated distribution (Figs 2C, 2E and 3A compared to 3F). A slower increase speed also relaxes the requirement of very high partner selectivity: the pattern found for a slow increase speed, intermediate decrease speed (LHW = 2880) and quite high partner selectivity (LPS = 0.95) also shows differentiated LIKE patterns. Differentiated patterns were also found for the comparable simulation runs with the alternative dynamics (Figs 2B, 2D, 2F and 3). Both the original and the alternative dynamics, in combination with a slower increase speed, result in a simulation run in which it takes more time to form a relationship. This leads to a LIKE distribution with a smaller number of high LIKE relationships. Furthermore, using the alternative dynamics, relationships are formed even more slowly while high LIKE relationships are maintained over a longer period of time. This results in fewer relationships that have intermediate relationship quality and a larger difference in average LIKE between intermediate and high quality relationships. Therefore, the differentiation caused by lowering the increase speed is much more pronounced for the alternative than the original dynamics.

Therefore, while both the original and alternative dynamics result in differentiated LIKE values in part of the partner selectivity (LPS) and speed of decrease (LHW) parameter space, they differ in how pronounced this differentiation is, especially when the increase speed is low (Figs 2 and 3). The original dynamics results in several relationships for each individual with LIKE values that tend to form a gradient from higher LIKE to lower LIKE, with higher LIKE values towards individuals that are closer in rank. The alternative dynamics in combination with a slow increase speed results in a situation where most relationships either tend to be close to 0 or close to 1. This difference between high-LIKE and low-LIKE relationships therefore is closer to a categorical difference as opposed to a continuous range as is the case for the original dynamics. While rank clearly plays a role, it cannot completely explain the distribution of LIKE. There are several cases in which an individual has a high LIKE score towards an individual that is not closest in rank, while having a lower LIKE score towards the individual that is closest in rank. The more pronounced differences between high-LIKE and low-LIKE relationships caused by the different dynamics are the result of grooming being distributed differently throughout the group, as opposed to an absolute difference in amount of time spent grooming, in any run in which most individuals have at least one high-LIKE relationship (S4 Fig).

### Similarity between LIKE, grooming and proximity

To investigate which parameter settings resulted in stable partner-specific grooming and proximity patterns, the same analysis that was used for the LIKE distributions was also performed on dyadic grooming and proximity distributions. For the original dynamics, the similarity of these patters has been established elsewhere [42, Fig 2]. Patterns of grooming, and less so proximity, are similar to the patterns of LIKE using the alternative dynamics and an intermediate increase speed (S5 Fig). This is also true for other parameter settings (see S6 and S7 Figs for an overview of all dyadic grooming and proximity distributions, respectively). Therefore, grooming and proximity also respond to changes in the dynamics, increase speed, decrease speed and selectivity in a way similar to LIKE. Differences in proximity between dyads are less clear than those for grooming and LIKE.

### Combinations of the original and alternative dynamics

**Fig 4:**
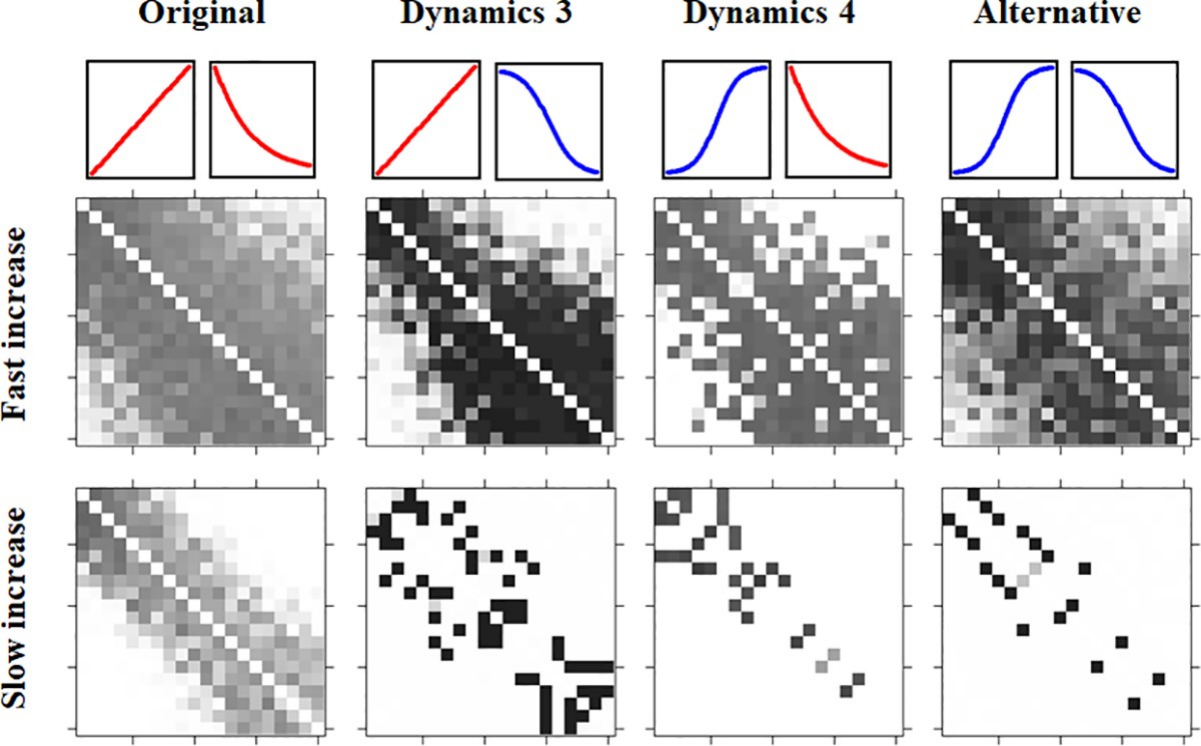
Dyadic LIKE values averaged over the “second year” of the recording period with a high level of partner selectivity (LPS) and an intermediate decrease speed (LHW = 2880) compared between the original and alternative dynamics as well as the two combined dynamics (dynamics 3 and dynamics 4) for two different increase speeds. On the y-axis individuals are ordered from low ranking (top row) to high ranking (bottom row). On the x-axis individuals are ordered from low ranking (left) to high ranking (right). Each square represents LIKE from one individual to another. LIKE ranges from 0.99 (black) to 0.01 (white). The simulation runs shown for the original dynamics and the alternative dynamics correspond to the two images on the right in Fig 3D and 3F.

We explored whether the differences found between the original and alternative dynamics were due to a difference in the way that LIKE increases or due to a difference in the way that LIKE decreases. To this end, two additional dynamics were investigated, combining the original increase dynamics with the alternative decrease dynamics (dynamics 3) and by combining the alternative increase dynamics with the original decrease dynamics (dynamics 4). These were compared to the original and the alternative dynamics (Fig 4). There is a visible difference when comparing the LIKE distributions that result from dynamics 3 and dynamics 4 to the original and alternative dynamics, showing that neither the change to the increase nor the change to the decrease of LIKE by itself causes the observed difference between the original and alternative dynamics. Both dynamics 3 and 4 are characterised by a larger difference between the highest and lowest LIKE values, since low LIKE values increase less quickly using dynamics 4 and high LIKE values decrease less quickly using dynamics 3 compared to the original dynamics. The two combined dynamics and the alternative dynamics, especially when combined with a slow increase speed, result in clear differentiation, with almost no relationships of intermediate relationship quality (see S8 Fig). The distributions that result using dynamics 3 and 4 seem to be clear intermediaries between the results of the alternative dynamics and the original dynamics.

### Stability of LIKE over time

**Fig 5:**
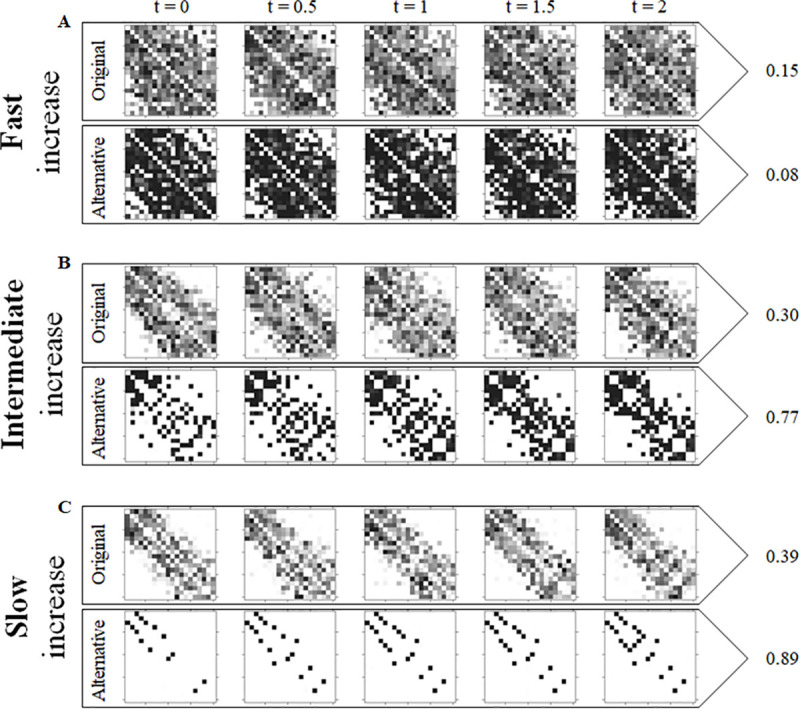
Stability of LIKE values over time for the original and the alternative dynamics for intermediate decrease speed (LHW = 2880), high partner selectivity (LPS = 0.99) and three different increase speeds. Each column shows dyadic LIKE values at a different point in time, from the LIKE distribution at the start of the recording period (t = 0) to the LIKE distribution at the end of the recording period (t = 2). An R^2^ value was calculated between adjacent moments in time and the 4 resulting R^2^ values were averaged. The average R^2^ is shown to the right of the 5 figures in each row. In each level plot individuals are ordered from low ranking (top row) to high ranking (bottom row) on the y-axis and from low ranking (left) to high ranking (right) on the x-axis. Each square represents LIKE from one individual to another. LIKE ranges from 0.99 (black) to 0.01 (white). These simulation runs correspond to the two images on the right in Fig 3D–3F.

To assess the stability of LIKE distributions over time, correlation scores were calculated between LIKE distributions at 5 different points in time and subsequently averaged (Fig 5). The average correlation for both the original and alternative dynamics in combination with a fast increase speed of LIKE is very low (R^2^ is 0.15 and 0.08, respectively; Fig 5A). The LIKE distribution is more stable when the increase speed is slower for both the original and alternative dynamics (Fig 5B). However, the average correlation of alternative dynamics in combination with a slow increase speed is more than twice as high as the average correlation of the original dynamics (R^2^ is respectively 0.89 and 0.39; Fig 5C).

Partner selectivity also has a strong influence on the stability of LIKE values (S9 Fig). A lower level of selectivity results in less stable relationships. A slightly lower partner selectivity (LPS = 0.9) will always result in a less stable LIKE distribution compared to the same parameter settings with very high partner selectivity (LPS = 0.99). However, the distribution of LIKE at lower selectively with the alternative dynamics is more stable than the distribution of LIKE with the original dynamics with high selectivity. This is also illustrated by a higher number of intermediate quality relationships in the original than alternative dynamics (see S2 Fig). Therefore, while the original dynamics require strong partner selectivity to form for stable relationships, the alternative dynamics with a slow increase speed and intermediate decrease speed still leads to stable relationships when partner selectivity is more relaxed.

## Discussion

This study investigated the effect of two different types of dynamics in combination with different speeds of change of an individual’s partner-specific valuation (LIKE) on the formation and maintenance of affiliative relationships, measured in the agent-based EMO-model that simulates primate behaviour. The original LIKE dynamics of the EMO-model resulted at group level in gradually differentiated affiliative relationships and at dyadic level in some very good relationships. The alternative dynamics resulted at group level in more differentiated and more stable affiliative relationships. These alternative dynamics, inspired by empirical data [43–46], resulted in more differentiated and more stable relationships. Differentiation and stability were higher for both dynamics when LIKE increased slowly. Thus, the formation and differentiation of affiliative relationships depend on the speed and manner of their increase during an affiliative interaction (in particular grooming) as well as their decrease during episodes without grooming.

The dynamics of the increase and decrease of LIKE have a clear effect on the differentiation of affiliative relationships, especially when the increase of LIKE is slow. The original dynamics [36] was not designed to promote the establishment and maintenance of affiliative relationships. In the original dynamics starting and strengthening of an affiliative relationship was independent of its current quality, but high LIKE values quickly diminished unless maintained through grooming interactions that followed each other quickly thanks to a very high partner selectivity. Therefore, only in a very small part of the parameter space these dynamics resulted in differentiated and long-term affiliative relationships [53]. The alternative dynamics was based on empirical results that indicate that starting an affiliative relationship is relatively difficult and maintaining it relatively easy. These dynamics led at a low increase speed to highly differentiated and very stable affiliative relationships, even when partner selectivity was less strong. Therefore, the dynamics of increase and decrease of LIKE have a clear influence on the distribution and stability of affiliative relationships in a group, and affect how differentiated these relationships are.

In addition, the speed of increase and decrease of LIKE is important. At low speeds of increase, relationships become more differentiated and stable. For both dynamics, the patterns in the distributions of LIKE, grooming and proximity show that an intermediate decrease speed and a high level of selectivity are both required for the formation of stable affiliative relationships. Moreover, this study shows that with a slower increase speed, a slower decrease speed is also required for stable affiliative relationships to arise. If it takes more time to form a social bond, the individuals have to remember these relationships longer as well if this relationship is not to be ‘forgotten’. While partner selectivity needed to be as high as possible when using the original dynamics, the alternative dynamics also results in stable and differentiated LIKE distributions when partner selectivity is lower. Thus, perhaps rather counter intuitively, when affiliative relationships are less easily formed, they are more stable.

The effect of different combinations of the original and alternative dynamics of increase and decrease were explored. The results of the simulation runs that used a combination of the alternative and the original dynamics were much more differentiated than those of the original dynamics. Since both combined dynamics resulted in less intermediate quality relationships than the original dynamics, both the alternative decrease dynamics (i.e. alternative dynamics and dynamics 3) as well as the alternative increase dynamics (i.e. alternative dynamics and dynamics 4) contribute to forming differentiated relationships. Counterintuitively, the slower initial increase speed of dynamics 4 leads to a larger number of high quality relationships compared to the original dynamics. Combined with a slow increase speed, dynamics 3 results in the highest number of high quality relationships of the 4 dynamics settings. This indicates that both the dynamics of the decrease as well as of the increase of LIKE can play a significant role in this process. Crucially, observed differences between dyads in grooming were the result of individuals distributing their grooming differently and not simply due to a difference in absolute grooming rates.

This finding that individuals should not appreciate the received grooming by someone else too quickly in order for a long-term relationship to arise is in line with empirical studies that inspired our alternative dynamics, both concerning stress and bonding (oxytocin). Appreciation of a new affiliative partner may be slow initially due to increased anxiety when approached by an unfamiliar individual [43,44]. This slow increase is also suggested by studies in wild chimpanzees. In chimpanzees, oxytocin concentrations following an affiliative interaction are consistently higher for dyads that have a stronger affiliative relationship [45,46]. Thus, both differentiated stress and bonding reactions that depend on the quality of an affiliative relationship may be involved in emotional bookkeeping.

The finding that strong affiliative relationships are stable over long time periods also agrees with empirical findings. Baboons tend to have few strong affiliative relationships that are stable over a long period of time, while a larger number of weak affiliative relationships may change from month to month [47,48]. The assumption implicit in the original LIKE dynamics, the notion that a high-LIKE relationship would need more maintenance than a low-LIKE relationship, therefore does not agree with these empirical findings. Both the outcome of the alternative dynamics and empirical data suggest that individuals do not constantly need to invest in the maintenance of a strong bond.

The formation of affiliative relationships on the basis of grooming interactions is not directly reflective of how, in the EMO-model, these interactions affect an individual’s internal state, i.e. satisfaction. The original dynamics follows the proposed effect of grooming on satisfaction: a linear increase due to a cumulative effect and a dose dependent decrease (an exponential decrease; [36]). When the increase and decrease follow the speed of increase and decrease of satisfaction in reaction to grooming, no high LIKE values are found [53; LHW = 180, Fig 2] and affiliative relationships are not formed. Only at lower speeds of decrease does the original dynamics yield long-term stable affiliative relationships. This implies that emotional bookkeeping does not simply follow satisfaction of interactions, but that interactions must be transformed into LIKE in a specific manner. This is also suggested by the current study, where not only the speed of decrease, but also both the alternative dynamics and a slow increase speed impact the formation of long-term stable LIKE distributions. Stable affiliative relationships are only formed when the dynamics and speed ensure that multiple grooming interactions are combined and their effect does not build up or fade away too quickly. This requires that such specific long-term accumulation of the effect of dyadic grooming interactions has been selected. The fitness benefits gained by strong affiliative relationships may lead to selection of favourable features, such as the alternative dynamics, a slow speed of increase and an intermediate speed of decrease retention. This also allows an individual to groom others than its most preferred partner or to cease grooming the preferred partner for a period of time. Thus, the alternative dynamics and a slow speed allow for a rich social life with preferred partners and attention to others.

Whether strongly differentiated and stable affiliative relationships are indeed found when friends are not easily won but also not easily lost, remains to be established empirically. The two main requirements that were identified by this study may be tested empirically. The concept that a slower formation of affiliative relationships should lead to more stable relationships and more pronounced differences between high- and low-quality relationships may be investigated within species as well as between different species. Within species, individuals that are slower to approach newcomers may develop fewer, but stronger and more stable relationships than individuals that easily contact newcomers, which would be in line with the idea that introverts tend to have fewer but stronger relationships than extroverts [31]. Between species, relationships are expected to be less differentiated in species where approaching newcomers is easy than in those where it is tense. Testing these predictions may be achieved by measuring their latency to interact with an unfamiliar individual. In addition, it can be investigated whether strong relationships require more maintenance than weak ones, or that indeed they are more easily maintained. For example, by investigating whether the strength of a relationship is associated with the longest period of time without interactions within a dyad, or by looking at the increase of stress (cf. [43,44]) or oxytocin (cf. [45,46]) that can be measured following an affiliative interaction in relation to the period without interactions. If an individual’s oxytocin response is measured after interacting with a preferred and a less preferred individual, this would give a potential measure of the difference in the increase speed of the emotional valuation. After not allowing the test subject to interact with either of these two partners for a set period of time, the oxytocin response is measured again for both dyads. If the difference between the strong and the weak relationship has become larger this would suggest that the stronger relationship is more stable over time.

## Conclusion

In conclusion, the dynamics and speed employed in emotional bookkeeping influence the ease with which strong affiliative relationships are formed and maintained. The alternative dynamics, implying difficulty to start or strengthen a new or weak relationship but ease to maintain a strong relationship as inspired by empirical data, gave rise to more strongly differentiated and more stable relationships over time compared to the original dynamics that was used in the 2015 paper by Evers et al [42]. Moreover, only when this process of emotional bookkeeping is rather strictly employed will strong stable affiliative relationships arise. Since the dynamics directly influences the quantity and quality of relationships in a group and since strong affiliative relationships may provide fitness benefits, this study suggests that the dynamics hinted at in empirical data has been selected to improve the formation of affiliative relationship through emotional bookkeeping. While this study did not observe actual animals, the model gave clear predictions that can be tested empirically. All in all, this modelling study shows that in a social group of primates a clear differentiation between friends and non-friends will arise when friends are not made too quickly and are remembered well.

## Supporting information

S1 Fig Dyadic LIKE values averaged over the “second year” of the recording period for four different levels of selectivity (LPS) and six different decrease speeds (LHW). On the y-axis individuals are ordered from low ranking (top row) to high ranking (bottom row). On the x-axis individuals are ordered from low ranking (left) to high ranking (right). Each square represents LIKE from one individual to another. LIKE ranges from 0.99 (black) to 0.01 (white). Figures **a**, **b** and **c** show the original dynamics with a fast, intermediate and slow increase speed respectively. Figures **d**, **e** and **f** show the alternative dynamics with a fast, intermediate and slow increase speed respectively.
(PDF)

S2 Fig The number of relationships categorised as high, intermediate and low quality for the original and alternative dynamics for the three increase speeds, four levels of partner selectivity (LPS) and intermediate decrease speed (LHW = 2880). Each bar represents all 380 dyadic relationships in a single simulation run.
(PDF)

S3 Fig The average LIKE value of relationships categorised as high, intermediate and low quality for the original and alternative dynamics for the three increase and six decrease speeds with very high partner selectivity (LPS = 0.99). The three different background colours correspond to high quality relationships (LIKE ≥ 0.75), intermediate quality relationships (0.25 < LIKE < 0.75) and low quality relationships (LIKE ≤ 0.25).
(PDF)

S4 Fig The group average of grooming for 6 different LHWs that were found in the 6 different combinations of dynamics and speed, i.e. the original dynamics and the alternative dynamics each with three different increase speeds. (PDF)

S5 Fig Dyadic LIKE values and dyadic grooming and proximity rates averaged over the “second year” of the recording period with alternative dynamics and intermediate increase speed, for two different levels of selectivity (LPS) and two different decrease speeds (LHW). On the y-axis individuals are ordered from low ranking (top row) to high ranking (bottom row). On the x-axis individuals are ordered from low ranking (left) to high ranking (right). Each square represents LIKE (**row 1**), grooming (**row 2**) or proximity (**row 3**) from one individual to another. LIKE ranges from 0.99 (black) to 0.01 (white). Grooming ranges from 9.1 (black) to 0 (white). Proximity ranges from 0.58 (black) to 0 (white). The figures show runs using the alternative dynamics and an intermediate increase speed. These simulation runs correspond to the bottom two images in Fig 3B and 3E. See for the comparison between dyadic grooming, proximity and LIKE-values with the original dynamics: [42, Fig 2].
(PDF)

S6 Fig Dyadic grooming rates averaged over the “second year” of the recording period for four different levels of selectivity (LPS) and six different decrease speeds (LHW). On the y-axis individuals are ordered from low ranking (top row) to high ranking (bottom row). On the x-axis individuals are ordered from low ranking (left) to high ranking (right). Each square represents grooming from one individual to another. Figures a, b and c show the original dynamics with a fast, intermediate and slow increase speed respectively. Figures d, e and f show the alternative dynamics with a fast, intermediate and slow increase speed respectively. Grooming ranges a: From 2.6 (black) to 0 (white); b: 3.4 to 0; c: 5.1 to 0; d: 2.6 to 0; e: 9.1 to 0; f: 11.1 to 0.
(PDF)

S7 Fig Dyadic proximity rates averaged over the “second year” of the recording period for four different levels of selectivity (LPS) and six different decrease speeds (LHW). On the y-axis individuals are ordered from low ranking (top row) to high ranking (bottom row). On the x-axis individuals are ordered from low ranking (left) to high ranking (right). Each square represents proximity from one individual to another. Figures **a**, **b** and **c** show the original dynamics with a fast, intermediate and slow increase speed respectively. Figures **d**, **e** and **f** show the alternative dynamics with a fast, intermediate and slow increase speed respectively. Proximity ranges from 0.4 (black) to 0 (white) in figures **a**, **b** and **c**: And from 0.58 to 0 in figures **d**, **e** and **f**.
(PDF)

S8 Fig The number of relationships categorised as high, intermediate and low quality for the original dynamics, dynamics 3, dynamics 4 and the alternative dynamics for two increase speeds (fast and slow), intermediate decrease speed (LHW = 2880) and very high partner selectivity (LPS = 0.99). Each bar represents all 380 dyadic relationships in a single simulation run.
(PDF)

S9 Fig Stability of LIKE values over time for the alternative dynamics, intermediate decrease speed (LHW = 2880) and four different levels of partner selectivity (LPS). Each column shows dyadic LIKE values at a different point in time, from the LIKE distribution at the start of the recording period (t = 0) to the LIKE distribution at the end of the recording period (t = 2). An R^2^ value was calculated between adjacent years and the 4 resulting R^2^ values were averaged. The average R^2^ is shown to the right of the 5 figures in each row. On the y-axis individuals are ordered from low ranking (top row) to high ranking (bottom row). On the x-axis individuals are ordered from low ranking (left) to high ranking (right). Each square represents LIKE from one individual to another. LIKE ranges from 0.99 (black) to 0.01 (white). Row 1 corresponds to the bottom row in Fig 5C and to the bottom right image in Fig 3F. Row 2 corresponds to the bottom left image in Fig 3F.
(PDF)

S1 Table Levels of steepness used for the different increase and decrease speeds of LIKE. (PDF)

## References

[1] Silk JB, Alberts SC, Altmann J (2003) Social Bonds of Female Baboons. Science, 302(November), 1231–1235. 10.1126/science.1088580 14615543

[2] Massen JJM, Sterck EHM, De Vos H (2010) Close social associations in animals and humans: Functions and mechanisms of friendship. Behaviour, 147(11), 1379–1412. 10.1163/000579510X528224.

[3] Seyfarth R. M., & Cheney D. L. (2012). The Evolutionary Origins of Friendship. Annual Review of Psychology, 63(1), 153–177. 10.1146/annurev-psych-120710-100337 21740224

[4] Wrangham R. W. (1980). An Ecological Model of Female-Bonded Primate Groups. Behaviour, 75(3–4).

[5] Silk J (2002). Using the “F”-word in primatology. Behaviour, 139(2), 421–446. 10.1163/156853902760102735.

[6] Silk JB, Alberts SC, Altmann J (2006a) Social relationships among adult female baboons (Papio cynocephalus) II. Variation in the quality and stability of social bonds. *Behavioral Ecology and Sociobiology*, 61(2), 197–204. 10.1007/s00265-006-0250-9.

[7] Silk JB, Altmann J, Alberts SC (2006b) Social relationships among adult female baboons (papio cynocephalus) I. Variation in the strength of social bonds. *Behavioral Ecology and Sociobiology*, 61(2), 183–195. 10.1007/s00265-006-0249-2.

[8] Massen JJM, Sterck EHM (2013) Stability and Durability of Intra- and Intersex Social Bonds of Captive Rhesus Macaques (*Macaca mulatta*). International Journal of Primatology, 34(4), 770–791. 10.1007/s10764-013-9695-7.

[9] Dunbar RIM (1980) Determinants and evolutionary consequences of dominance among female gelada baboons. Behavioral Ecology and Sociobiology, 7(4), 253–265. 10.1007/BF00300665.

[10] Schino G, Aureli F (2009) Reciprocal Altruism in Primates. Partner Choice, Cognition, and Emotions. Advances in the Study of Behavior (1st ed., Vol. 39). Elsevier Inc. 10.1016/S0065-3454(09)39002-6.

[11] Gomes CM, Mundry R, Boesch C (2009) Long-term reciprocation of grooming in wild West-African chimpanzees. Proceedings of the Royal Society B: Biological Sciences 276:699–706 10.1098/rspb.2008.1324 18957365 PMC2660945

[12] Mitani JC (2009) Male chimpanzees form enduring and equitable social bonds. Animal Behaviour 77:633–640 10.1016/j.anbehav.2008.11.021

[13] Langergraber K, Mitani J, Vigilant L (2009) Kinship and social bonds in female chimpanzees (Pan troglodytes). American Journal of Primatology 71:840–851 10.1002/ajp.20711 19475543

[14] Berghänel A, Ostner J, Schröder U, Schülke O (2011) Social bonds predict future cooperation in male Barbary macaques, *Macaca sylvanus*. Animal Behaviour 81:1109–1116 10.1016/j.anbehav.2011.02.009

[15] Archie EA, Moss CJ, Alberts SC (2006) The ties that bind: genetic relatedness predicts the fission and fusion of social groups in wild African elephants. Proceedings of the Royal Society B: Biological Sciences 273:513–522 10.1098/rspb.2005.3361 16537121 PMC1560064

[16] Connor RC, Wells RS, Mann J, Read AJ (2000) The bottlenose dolphin. In: Mann J, Connor RC, Tyack PL, Whitehead H, eds. Cetacean Societies. Field studies of dolphins and whales, Chicago: University of Chicago Press, 91–125.

[17] Connor RC, Heithaus MR, Barre LM (2001) Complex social structure, alliance stability and mating access in a bottlenose dolphin ‘‘super-alliance”. Proceedings of the Royal Society of London. Series B: Biological Sciences 268:263–267 10.1098/rspb.2000.1357 11217896 PMC1088601

[18] Wiszniewski J, Lusseau D, Möller LM (2010) Female bisexual kinship ties maintain social cohesion in a dolphin network. Animal Behaviour 80:895–904 10.1016/j.anbehav.2010.08.013

[19] Wiszniewski J, Brown C, Möller LM (2012) Complex patterns of male alliance formation in a dolphin social network. Journal of Mammalogy 93:239–250 10.1644/10-MAMM-A-366.1

[20] Weidt A, Hofmann SE, König B (2008) Not only mate choice matters: fitness consequences of social partner choice in female house mice. Animal Behaviour, 75(3), 801–808. 10.1016/j.anbehav.2007.06.017.

[21] Silk JB, Beehner JC, Bergman TJ, Crockford C, Engh AL, Moscovice LR, et al (2009) The benefits of social capital: close social bonds among female baboons enhance offspring survival. *Proceedings of the Royal Society B*: *Biological Sciences*, 276(1670), 3099–3104. 10.1098/rspb.2009.0681 19515668 PMC2817129

[22] Frère CH, Krutzen M, Mann J, Connor RC, Bejder L, Sherwin WB (2010) Social and genetic interactions drive fitness variation in a free-living dolphin population. Proceedings of the National Academy of Sciences, 107(46), 19949–19954. 10.1073/pnas.1007997107. PMC2993384 21041638

[23] Schülke O, Bhagavatula J, Vigilant L, Ostner J (2010) Social bonds enhance reproductive success in male macaques. Current Biology, 20(24), 2207–2210. 10.1016/j.cub.2010.10.058 21093261

[24] Silk JB, Beehner JC, Bergman TJ, Crockford C, Engh AL, Moscovice LR, et al (2010a) Strong and consistent social bonds enhance the longevity of female baboons. Current Biology 20:1359–1361 10.1016/j.cub.2010.05.067 20598541

[25] Archie EA, Tung J, Clark M, Altmann J, Alberts SC (2014) Social affiliation matters: both same-sex and opposite-sex relationships predict survival in wild female baboons. Proceedings of the Royal Society B: Biological Sciences, 281(1793), 20141261–20141261. 10.1098/rspb.2014.1261 25209936 PMC4173677

[26] Dunbar RIM (2018) Social structure as a strategy to mitigate the costs of group living: a comparison of gelada and guereza monkeys. Animal Behaviour, 136, 53–64. 10.1016/j.anbehav.2017.12.005 29497179 PMC5825386

[27] Dunbar RIM (2018) The Anatomy of Friendship. Trends in Cognitive Sciences, 22(1), 32–51. 10.1016/j.tics.2017.10.004 29273112

[28] Saramäki J, Leicht EA, López E, Roberts SGB, Reed-Tsochas F, Dunbar RIM (2014) Persistence of social signatures in human communication. Proceedings of the National Academy of Sciences of the United States of America, 111(3), 942–947. 10.1073/pnas.1308540110 24395777 PMC3903242

[29] Sutcliffe A, Wang D (2012) Computational modelling of trust and social relationships. Jasss, 15(1). 10.18564/jasss.1912.

[30] Sutcliffe AG, Dunbar RIM, Wang D (2016) Modelling the evolution of social structure. PLoS ONE, 11(7), 10–16. 10.1371/journal.pone.0158605 27427758 PMC4948869

[31] Tamarit I, Cuesta JA, Dunbar RIM, Sánchez A (2018) Cognitive resource allocation determines the organization of personal networks. Proceedings of the National Academy of Sciences of the United States of America, 115(33), 8316–8321. 10.1073/pnas.1719233115 30049707 PMC6099867

[32] Puga-Gonzalez I, Hoscheid A, Hemelrijk CK (2015) Friendships, reciprocation and interchange in an individual-based model. Behavioral Ecology and Sociobiology 69:383_394 10.1007/s00265-014-1850-4

[33] De Waal FBM, Luttrell LM (1988) Mechanisms of social reciprocity in three primate species: Symmetrical relationship characteristics or cognition? Ethology and Socio- biology 9:101–118 10.1016/0162-3095(88)90016-7

[34] Aureli F, Schaffner CM (2002) Relationship assessment through emotional mediation. Behaviour 139:393–420 10.1163/156853902760102726

[35] Aureli F, Schino G (2004) The role of emotions in social relationships. In: Thierry B, Singh M, Kaumanns W, eds. Macaque Societies: a model for the study of social organization. Vol. 41. Cambridge: Cambridge University Press, 38–55.

[36] Evers E, De Vries H, Spruijt BM, Sterck EHM (2014) The EMO-model: an agent-based model of primate social behavior regulated by two emotional dimensions, anxiety-FEAR and satisfaction-LIKE. PLoS ONE 9:e87955 10.1371/journal.pone.0087955 24504194 PMC3913693

[37] Cheney DL, Seyfarth RM (1990) The representation of social relations by monkeys. Cognition 37:167–196 10.1016/0010-0277(90)90022-c 2269006

[38] Kummer H, Götz W, Angst W (1974) Triadic differentiation: an inhibitory process protecting pair bonds in baboons. Behaviour 49:62–87 10.1163/156853974x00408 4208112

[39] Cheney DL, Seyfarth RM (1986) The recognition of social alliances by vervet monkeys. Animal Behaviour 34:1722–1731 10.1016/S0003-3472(86)80259-7

[40] Dasser V (1988) A social concept in Java monkeys. Animal Behaviour 36:225–230 10.1016/S0003-3472(88)80265-3

[41] Bergman TJ, Beehner JC, Cheney DL, Seyfarth RM (2003) Hierarchical classification by rank and kinship in baboons. Science 302:1234–1236 10.1126/science.1087513 14615544

[42] Evers E, De Vries H, Spruijt BM, Sterck EHM (2015) Emotional bookkeeping and high partner selectivity are necessary for the emergence of partner-specific reciprocal affiliation in an agent-based model of primate groups. PLoS ONE 10:e0118921 10.1371/journal.pone.0118921 25785601 PMC4364990

[43] Young C, Majolo B, Heistermann M, Schülke O, Ostner J (2014). Responses to social and environmental stress are attenuated by strong male bonds in wild macaques. Proceedings of the National Academy of Sciences, 111(51), 18195–18200. 10.1073/pnas.1411450111 25489097 PMC4280642

[44] Aureli F, Preston SD, De Waal FBM (1999) Heart rate responses to social interactions in free-moving rhesus macaques (Macaca mulatta): a pilot study. Journal of Compara- tive Psychology 113:59–65 10.1037/0735-7036.113.1.59 10098269

[45] Crockford C, Wittig RM, Langergraber K, Ziegler TE, Zuberbühler K, Deschner T (2013) Urinary oxytocin and social bonding in related and unrelated wild chim- panzees. Proceedings of the Royal Society B: Biological Sciences 280: 20122765 10.1098/rspb.2012.2765 23345575 PMC3574389

[46] Wittig RM, Crockford C, Deschner T, Langergraber KE, Ziegler TE, Zuberbühler K (2014) Food sharing is linked to urinary oxytocin levels and bonding in related and unrelated wild chimpanzees. Proceedings of the Royal Society B: Biological Sciences 281: 20133096 10.1098/rspb.2013.3096 24430853 PMC3906952

[47] Silk JB, Beehner JC, Bergman TJ, Crockford C, Engh AL, Moscovice LR, et al (2010b) Female chacma baboons form strong, equitable, and enduring social bonds. Behavioral Ecology and Sociobiology 64:1733–1747 10.1007/s00265-010-0986-0 20976293 PMC2952770

[48] Silk JB, Alberts SC, Altmann J, Cheney DL, Seyfarth RM (2012) Stability of partner choice among female baboons. Animal Behaviour 83:1511–1518 10.1016/j.anbehav.2012.03.028 23885128 PMC3718070

[49] Surbeck M, Hohmann G (2014). Social preferences influence the short-term exchange of social grooming among male bonobos. Animal Cognition, 18(2), 573–579. 10.1007/s10071-014-0826-0 25519436

[50] Hogeweg P, Hesper B (1979) Heterarchical, selfstructuring simulation systems: concepts and applications in biology. Methodologies in systems modelling and simulation. Amsterdam: North-Holland Publishing Company.

[51] Bryson JJ, Ando Y, Lehmann H (2007) Agent-based modelling as scientific method: a case study analysing primate social behaviour. Philosophical Transactions of the Royal Society B: Biological Sciences 362:1685–1699 10.1098/rstb.2007.2061 17434852 PMC2440780

[52] Campennì M, Schino G (2014) Partner choice promotes cooperation: the two faces of testing with agent-based models. Journal of Theoretical Biology 344:49_55 10.1016/j.jtbi.2013.11.019 24316108

[53] Evers E, De Vries H, Spruijt BM, Sterck EHM (2016) Intermediate-term emotional bookkeeping is necessary for long-term reciprocal grooming partner preferences in an agent-based model of macaque groups. PeerJ, 4, e1488. 10.7717/peerj.1488 26839737 PMC4734454

[54] Grimm V, Berger U, DeAngelis DL, Polhill JG, Giske J, Railsback SF (2010) The ODD protocol: a review and first update. Ecological Modelling 221:2760–2768 10.1016/j.ecolmodel.2010.08.019

[55] R Core Team (2015). R: A language and environment for statistical computing. R Foundation for Statistical Computing, Vienna, Austria. URL https://www.R-project.org/.

