# Supplementary material for: Emotional bookkeeping and differentiated affiliative relationships: Exploring the role of dynamics and speed in updating relationship quality in the EMO-model
Source: PLoS One. 2021 Apr 2;16(4):e0249519. doi: 10.1371/journal.pone.0249519 (PMC8018660; doi:10.1371/journal.pone.0249519)
Supplement: S1 Fig — On the y-axis individuals are ordered from low ranking (top row) to high ranking (bottom row). On the x-axis individuals are ordered from low ranking (left) to high ranking (right). Each square represents LIKE from one individual to another. LIKE ranges from 0.99 (black) to 0.01 (white). Figures a, b and c show the original dynamics with a fast, intermediate and slow increase speed respectively. Figures d, e and f show the alternative dynamics with a fast, intermediate and slow increase speed respectively. (PDF) [file pone.0249519.s001.pdf]

Emotional bookkeeping and differentiated affiliative relationships: exploring the role of dynamics and speed in updating relationship quality in the EMO-model

Tonko W Zijlstra, Han de Vries & Elisabeth HM Sterck

Supporting information S2: Dyadic LIKE values

Fig S2: Dyadic LIKE values averaged over the second year of the recording period for four different levels of selectivity (LPS) and six different decrease speeds (LHW). On the y-axis individuals are ordered from low ranking (top row) to high ranking (bottom row). On the x-axis individuals are ordered from low ranking (left) to high ranking (right). Each square represents LIKE from one individual to another. LIKE ranges from 0.99 (black) to 0.01 (white). Figures **a**, **b** and **c** show the original dynamics with a fast, intermediate and slow increase speed respectively. Figures **d**, **e** and **f** show the alternative dynamics with a fast, intermediate and slow increase speed respectively.

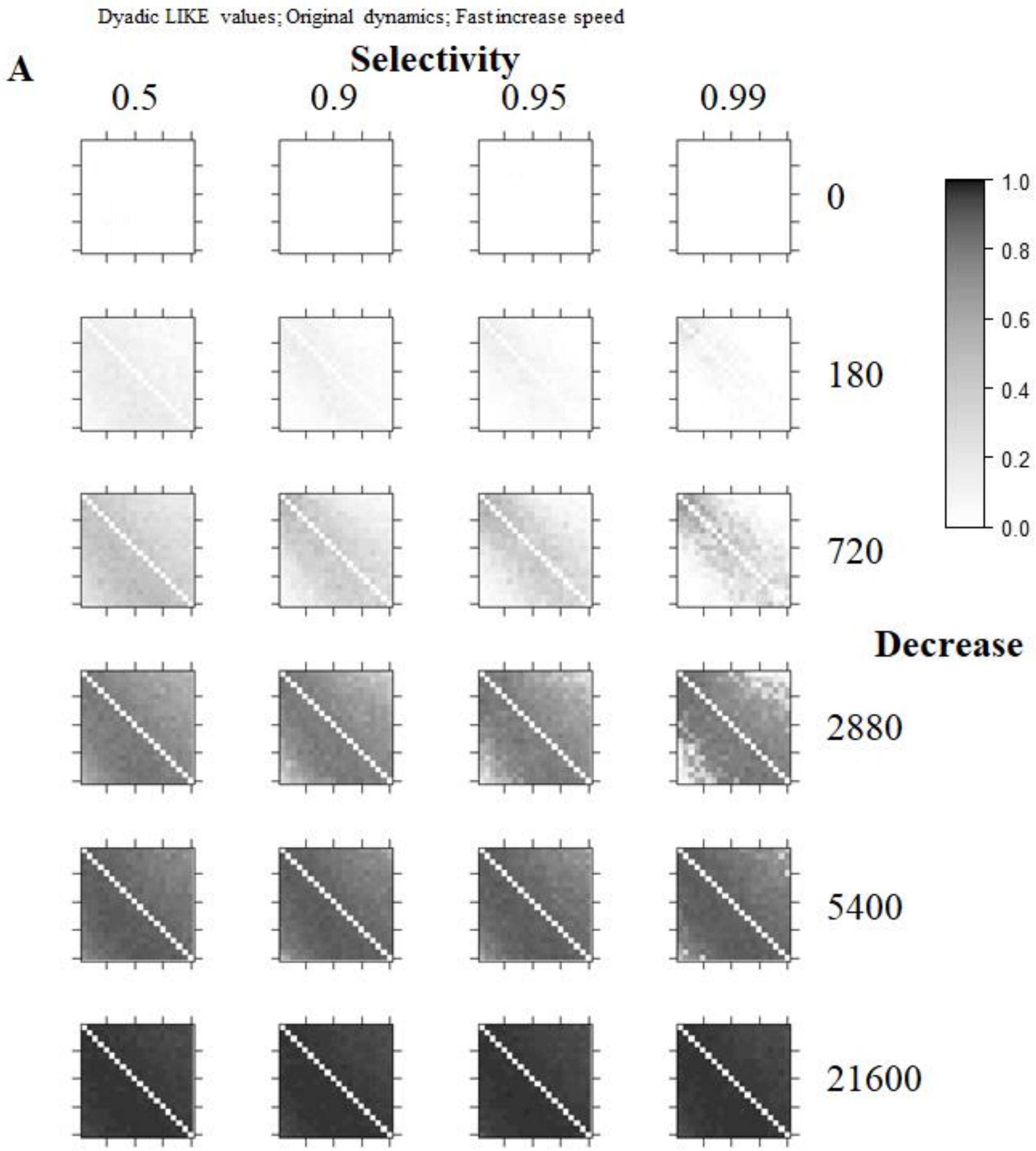

Dyadic LIKE values; Original dynamics; Fast increase speed
A
Selectivity
0.5
0.9
0.95
0.99
0
180
720
2880
5400
21600
Decrease
1.0
0.8
0.6
0.4
0.2
0.0

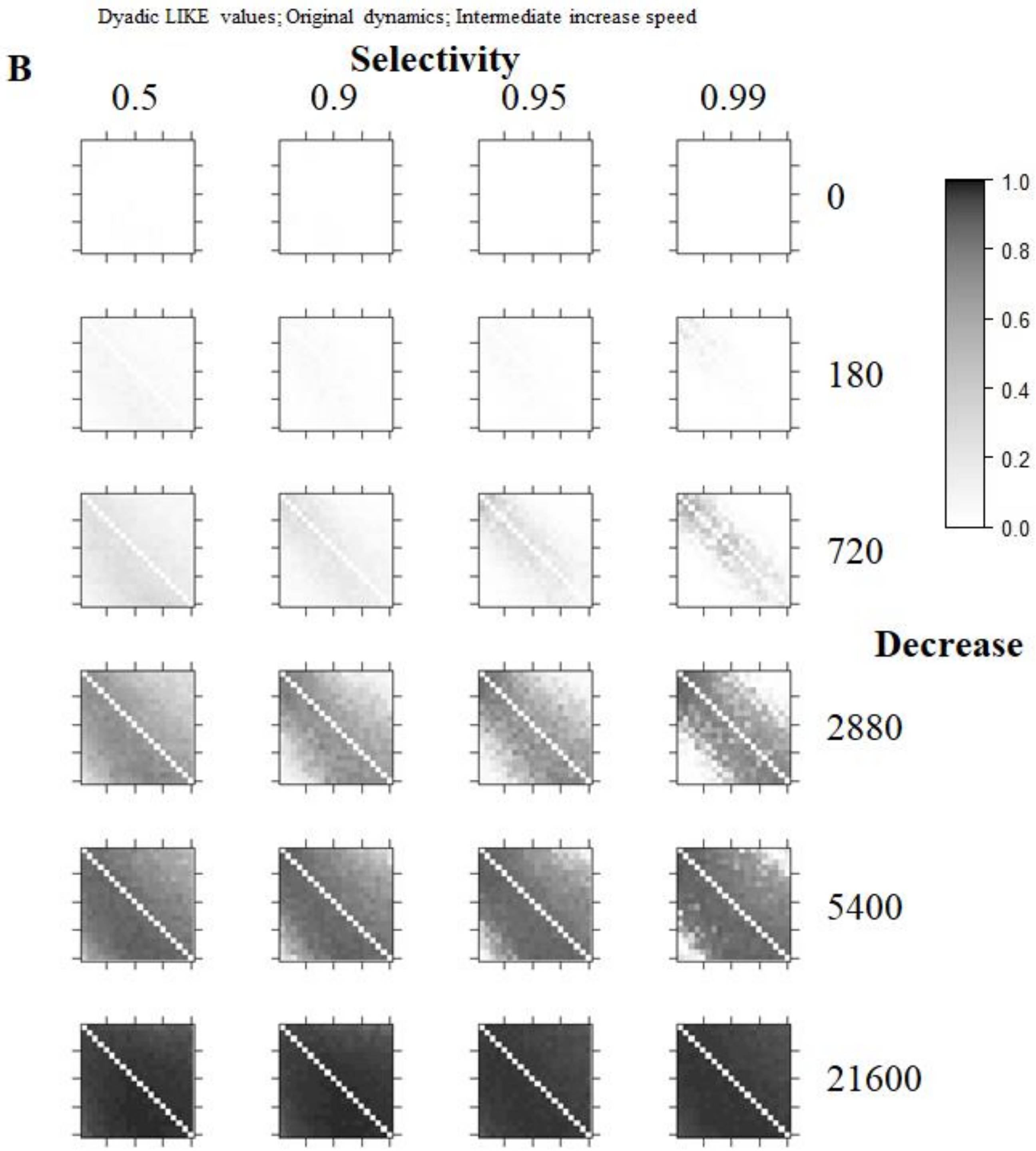
Dyadic LIKE values; Original dynamics; Intermediate increase speed
B
Selectivity
0.5
0.9
0.95
0.99
0
180
720
2880
5400
21600
Decrease
1.0
0.8
0.6
0.4
0.2
0.0

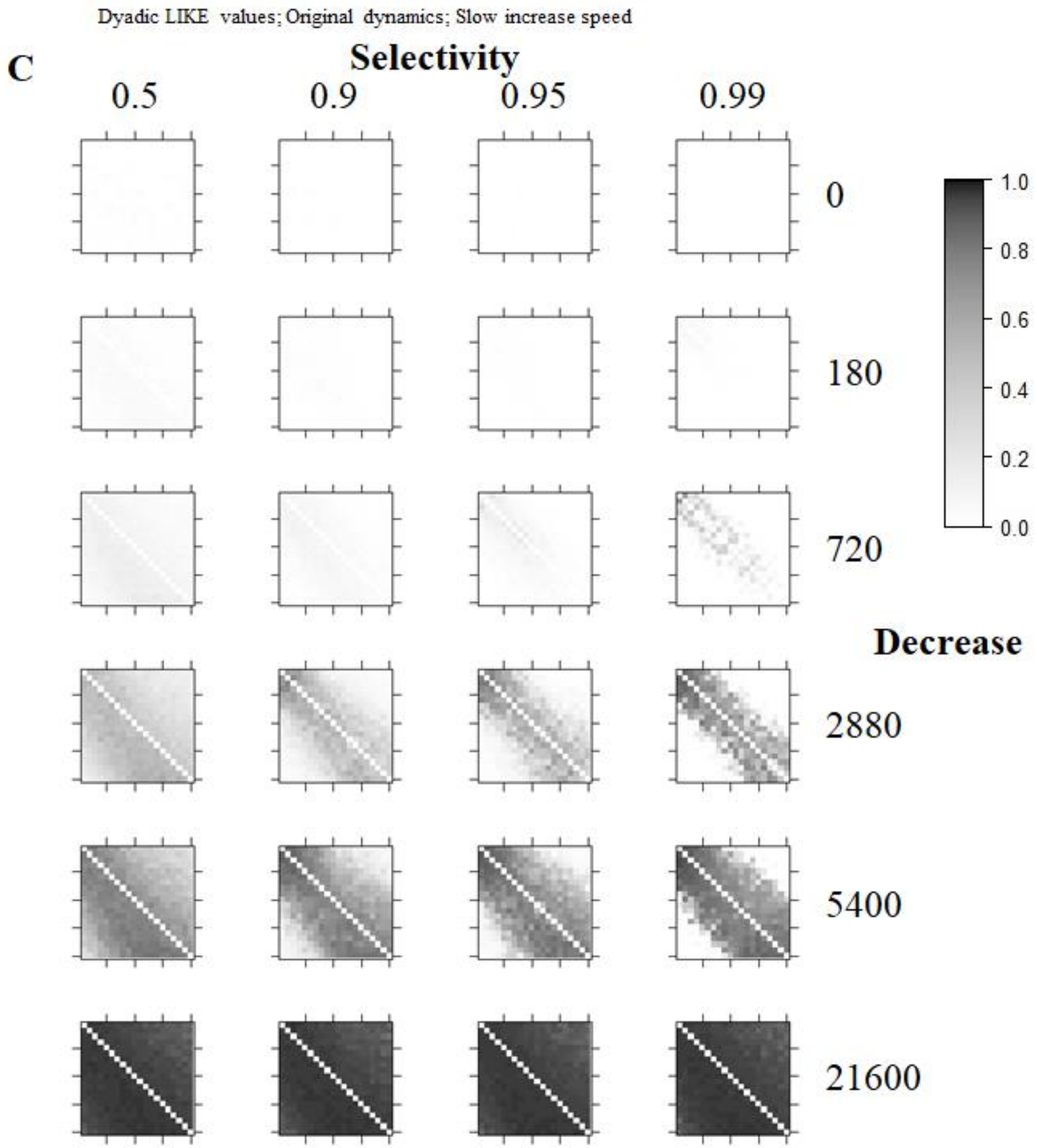
Dyadic LIKE values; Original dynamics; Slow increase speed
C
Selectivity
0.5
0.9
0.95
0.99
0
180
720
2880
5400
21600
Decrease
1.0
0.8
0.6
0.4
0.2
0.0

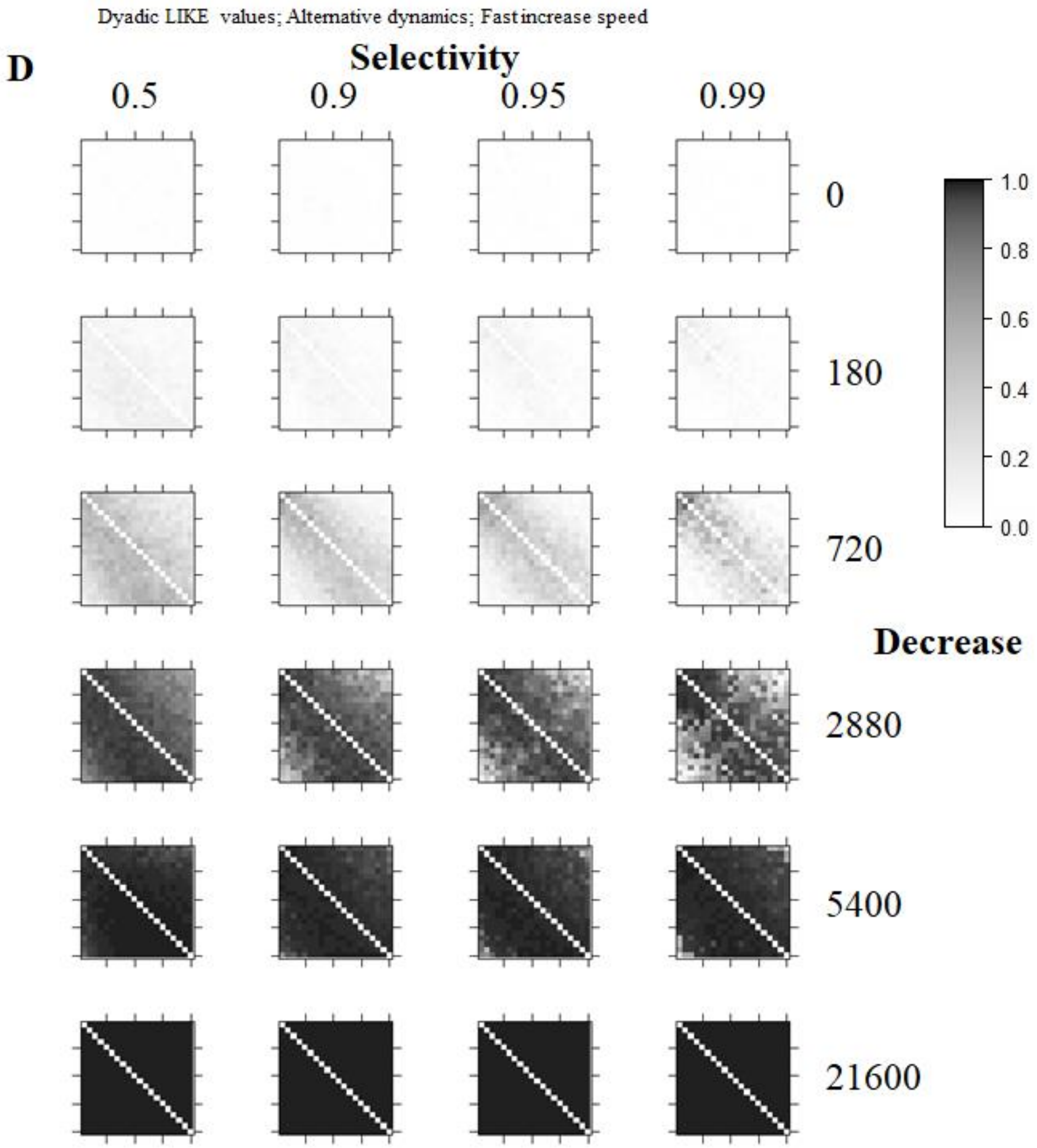
Dyadic LIKE values; Alternative dynamics; Fast increase speed
D
Selectivity
0.5
0.9
0.95
0.99
0
180
720
Decrease
2880
5400
21600
1.0
0.8
0.6
0.4
0.2
0.0

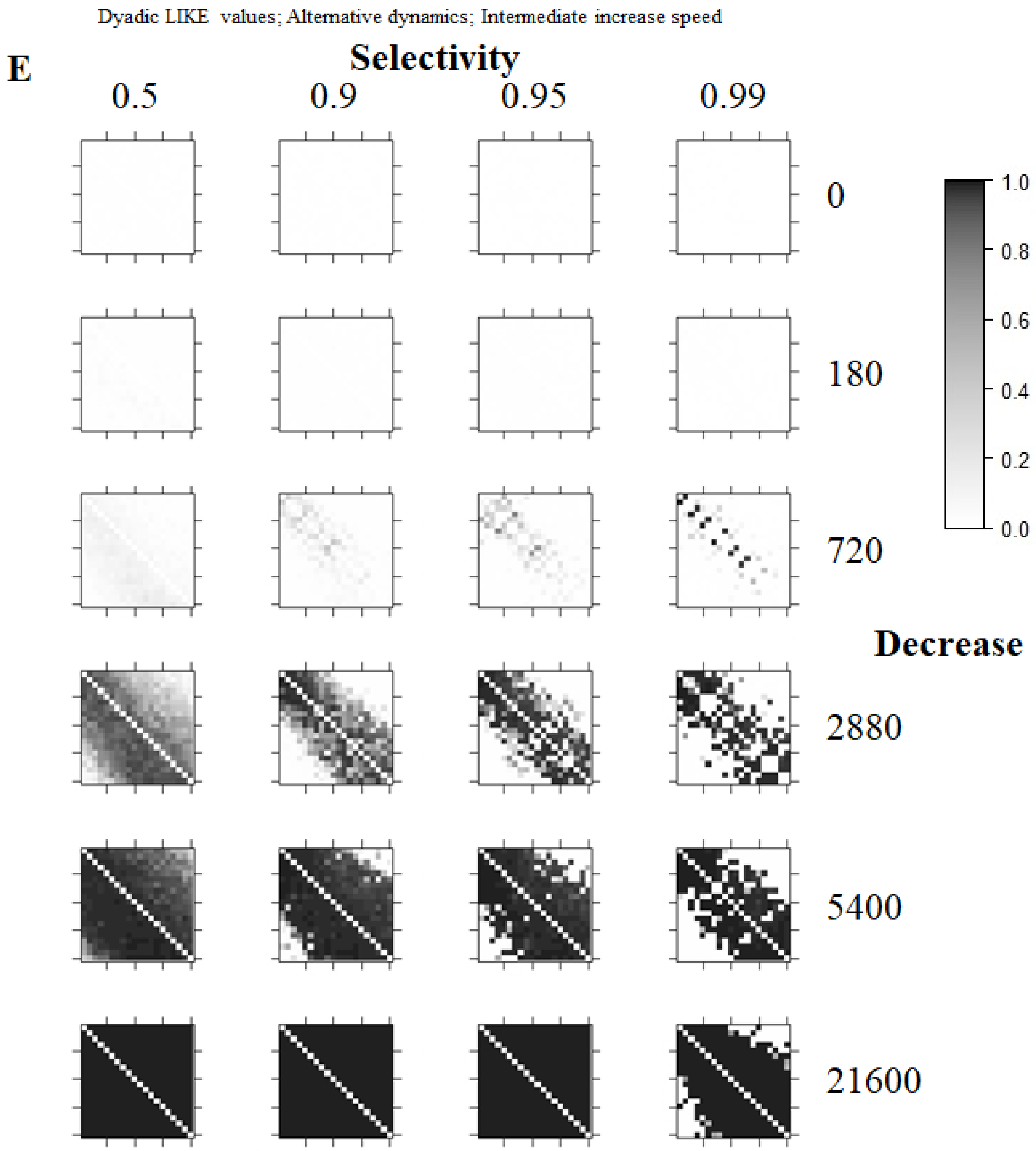

Dyadic LIKE values; Alternative dynamics; Intermediate increase speed
E
Selectivity
0.5
0.9
0.95
0.99
0
180
720
2880
5400
21600
Decrease
1.0
0.8
0.6
0.4
0.2
0.0

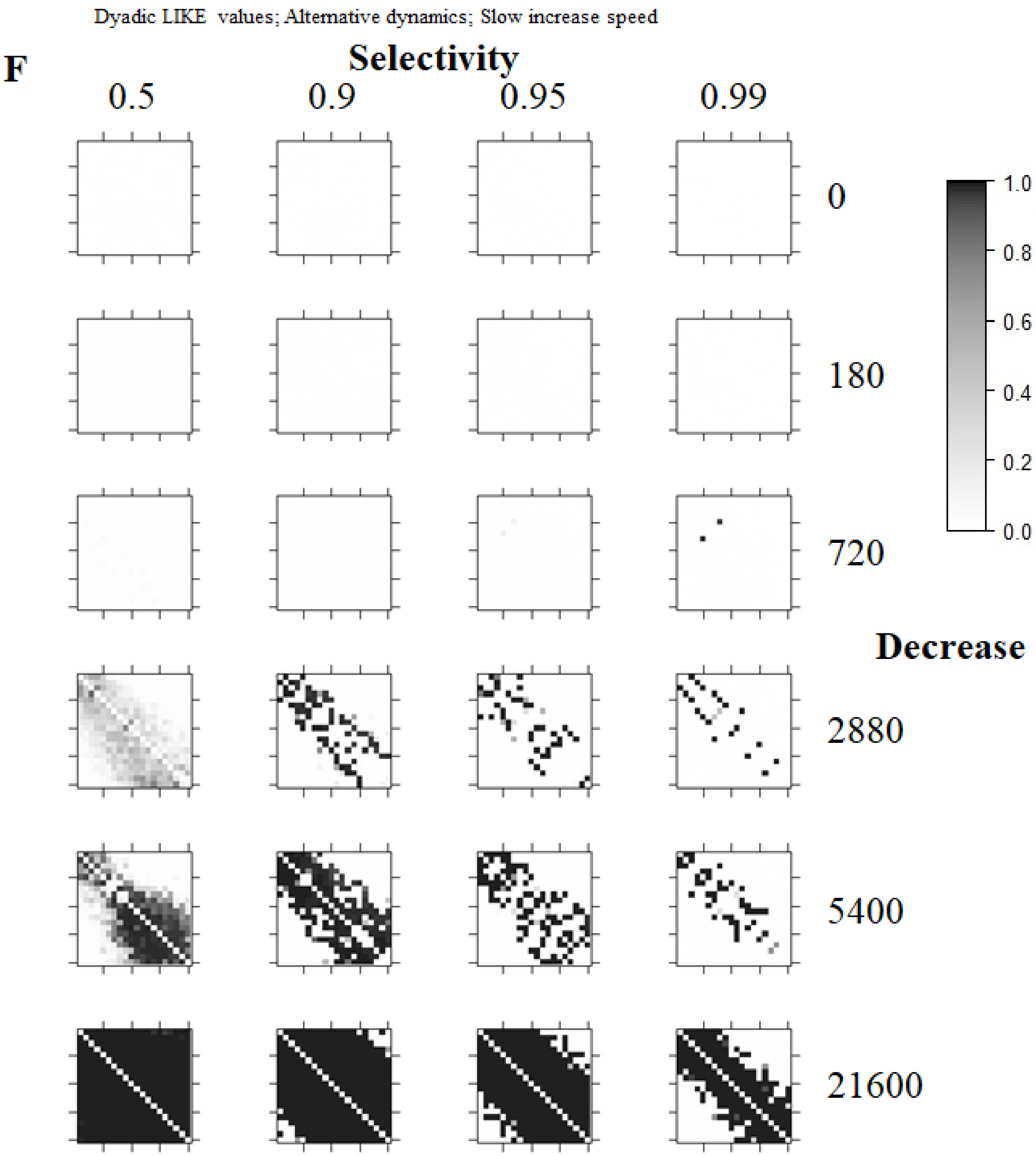
Dyadic LIKE values; Alternative dynamics; Slow increase speed
F
Selectivity
0.5
0.9
0.95
0.99
Decrease
0
180
720
2880
5400
21600
1.0
0.8
0.6
0.4
0.2
0.0
