# Supplementary material for: Emotional bookkeeping and differentiated affiliative relationships: Exploring the role of dynamics and speed in updating relationship quality in the EMO-model
Source: PLoS One. 2021 Apr 2;16(4):e0249519. doi: 10.1371/journal.pone.0249519 (PMC8018660; doi:10.1371/journal.pone.0249519)
Supplement: S2 Fig — Each bar represents all 380 dyadic relationships in a single simulation run. (PDF) [file pone.0249519.s002.pdf]

Emotional bookkeeping and differentiated affiliative relationships: exploring the role of dynamics and speed in updating relationship quality in the EMO-model

Tonko W Zijlstra, Han de Vries & Elisabeth HM Sterck

Supporting information S3: Impact of partner selectivity on differentiation

Fig S3: The number of relationships categorised as high, intermediate and low quality for the original and alternative dynamics for the three increase speeds, four levels of partner selectivity (LPS) and intermediate decrease speed (LHW=2880). Each bar represents all 380 dyadic relationships in a single simulation run.

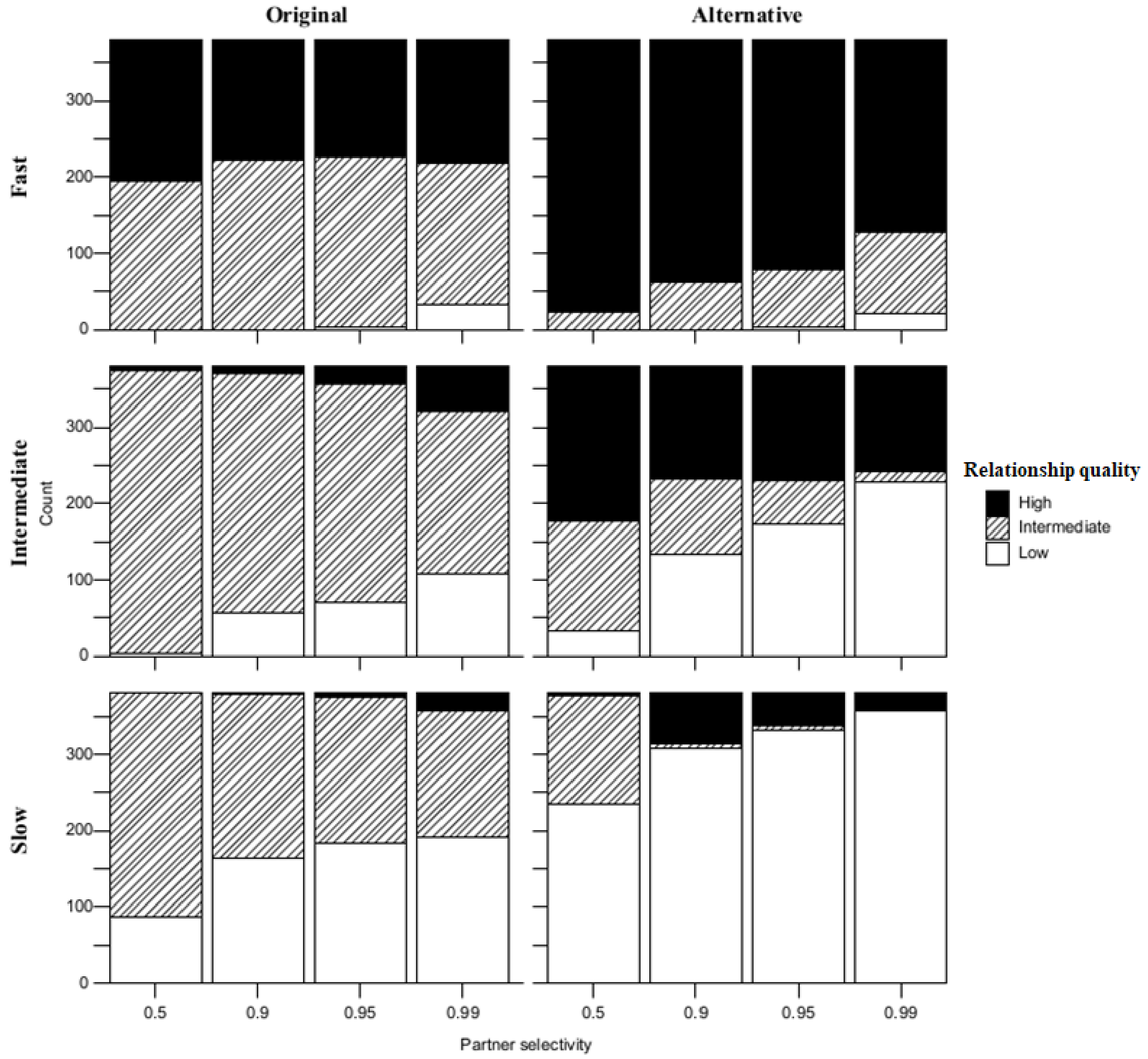

Original
Alternative
Fast
Intermediate
Slow
Count
0
100
200
300
Relationship quality
High
Intermediate
Low
0.5
0.9
0.95
0.99
Partner selectivity
