# Supplementary material for: Emotional bookkeeping and differentiated affiliative relationships: Exploring the role of dynamics and speed in updating relationship quality in the EMO-model
Source: PLoS One. 2021 Apr 2;16(4):e0249519. doi: 10.1371/journal.pone.0249519 (PMC8018660; doi:10.1371/journal.pone.0249519)
Supplement: S3 Fig — The three different background colours correspond to high quality relationships (LIKE ≥ 0.75), intermediate quality relationships (0.25 < LIKE < 0.75) and low quality relationships (LIKE ≤ 0.25). (PDF) [file pone.0249519.s003.pdf]

Emotional bookkeeping and differentiated affiliative relationships: exploring the role of dynamics and speed in updating relationship quality in the EMO-model

Tonko W Zijlstra, Han de Vries & Elisabeth HM Sterck

Supporting information S4: Average LIKE of relationship types

Fig S4: The average LIKE value of relationships categorised as high, intermediate and low quality for the original and alternative dynamics for the three increase and six decrease speeds with very high partner selectivity (LPS=0.99). The three different background colours correspond to high quality relationships (LIKE $\geq$ 0.75), intermediate quality relationships (0.25 $<$ LIKE $<$ 0.75) and low quality relationships (LIKE $\leq$ 0.25).

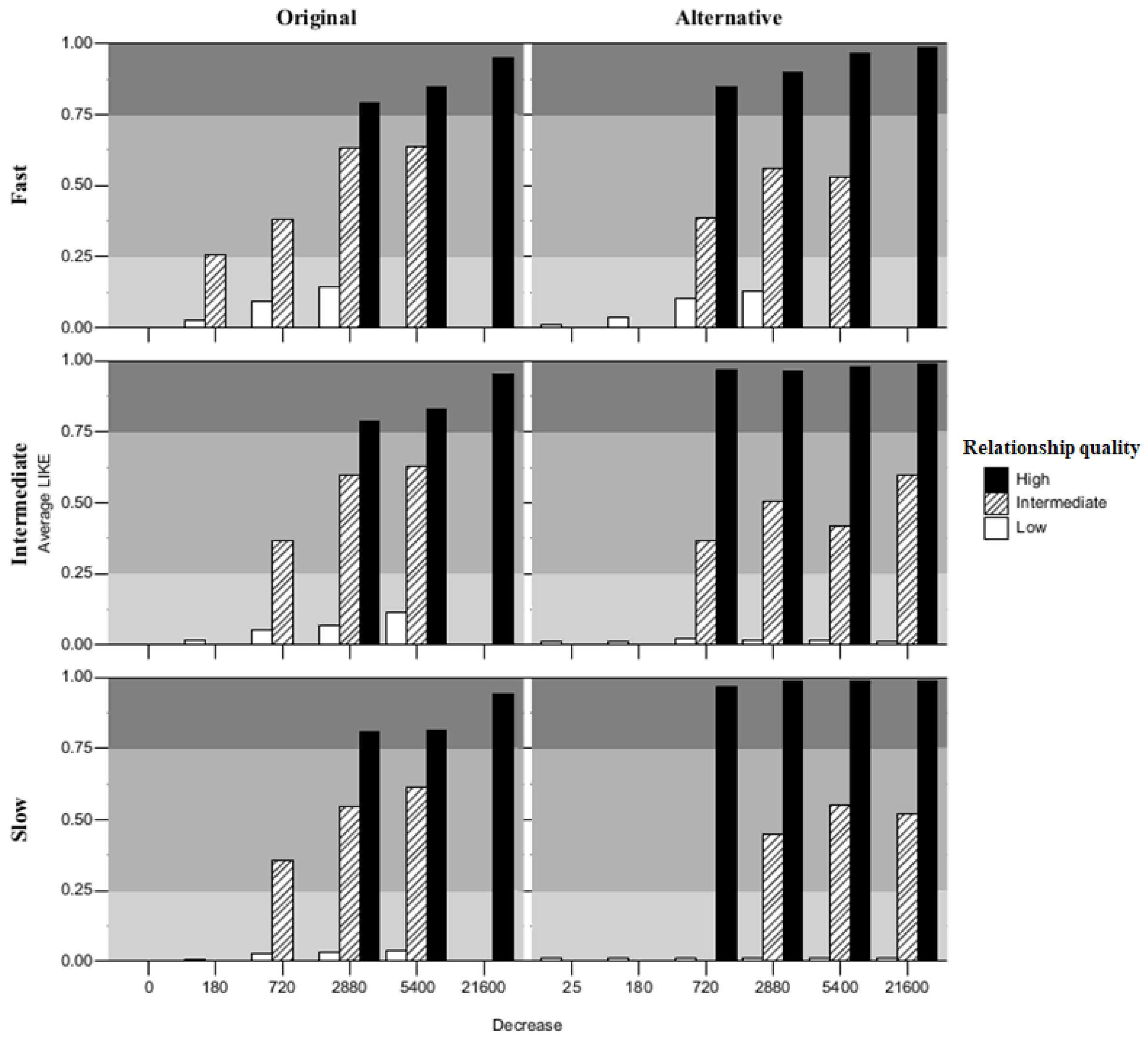

Original
Alternative
Fast
Intermediate
Slow
Average LIKE
1.00
0.75
0.50
0.25
0.00
0
180
720
2880
5400
21600
25
180
720
2880
5400
21600
Decrease
Relationship quality
High
Intermediate
Low
