# Supplementary material for: Emotional bookkeeping and differentiated affiliative relationships: Exploring the role of dynamics and speed in updating relationship quality in the EMO-model
Source: PLoS One. 2021 Apr 2;16(4):e0249519. doi: 10.1371/journal.pone.0249519 (PMC8018660; doi:10.1371/journal.pone.0249519)
Supplement: S4 Fig — (PDF) [file pone.0249519.s004.pdf]

Emotional bookkeeping and differentiated affiliative relationships: exploring the role of dynamics and speed in updating relationship quality in the EMO-model

Tonko W Zijlstra, Han de Vries & Elisabeth HM Sterck

Supporting information S5: Group average grooming rates

Fig S5: The group average of grooming for 6 different LHWs that were found in the 6 different combinations of dynamics and speed, i.e. the original dynamics and the alternative dynamics each with three different increase speeds.

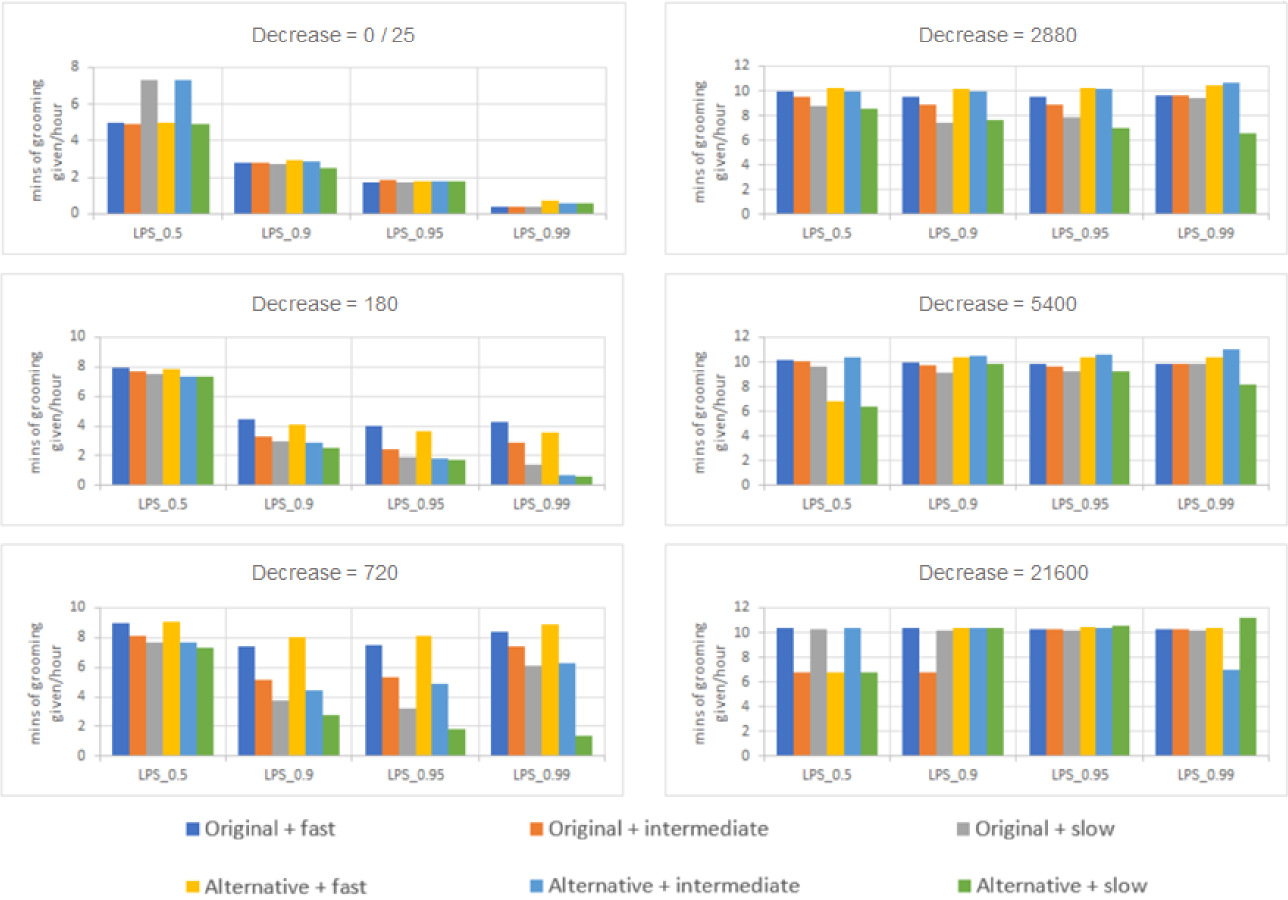
Decrease = 0 / 25
Decrease = 2880
Decrease = 180
Decrease = 5400
Decrease = 720
Decrease = 21600
mins of grooming given/hour
LPS_0.5
LPS_0.9
LPS_0.95
LPS_0.99
Original + fast
Original + intermediate
Original + slow
Alternative + fast
Alternative + intermediate
Alternative + slow
