# Supplementary material for: Emotional bookkeeping and differentiated affiliative relationships: Exploring the role of dynamics and speed in updating relationship quality in the EMO-model
Source: PLoS One. 2021 Apr 2;16(4):e0249519. doi: 10.1371/journal.pone.0249519 (PMC8018660; doi:10.1371/journal.pone.0249519)
Supplement: S5 Fig — On the y-axis individuals are ordered from low ranking (top row) to high ranking (bottom row). On the x-axis individuals are ordered from low ranking (left) to high ranking (right). Each square represents LIKE (row 1), grooming (row 2) or proximity (row 3) from one individual to another. LIKE ranges from 0.99 (black) to 0.01 (white). Grooming ranges from 9.1 (black) to 0 (white). Proximity ranges from 0.58 (black) to 0 (white). The figures show runs using the alternative dynamics and an intermediate increase speed. These simulation runs correspond to the bottom two images in Fig 3B and 3E. See for the comparison between dyadic grooming, proximity and LIKE-values with the original dynamics: [42, Fig 2]. (PDF) [file pone.0249519.s005.pdf]

Emotional bookkeeping and differentiated affiliative relationships: exploring the role of dynamics and speed in updating relationship quality in the EMO-model

Tonko W Zijlstra, Han de Vries & Elisabeth HM Sterck

Supporting information S6: Comparison between dyadic LIKE values and dyadic grooming and proximity rates

Fig S6: Dyadic LIKE values and dyadic grooming and proximity rates averaged over the "second year" of the recording period with alternative dynamics and intermediate increase speed, for two different levels of selectivity (LPS) and two different decrease speeds (LHW). On the y-axis individuals are ordered from low ranking (top row) to high ranking (bottom row). On the x-axis individuals are ordered from low ranking (left) to high ranking (right). Each square represents LIKE (**row 1**), grooming (**row 2**) or proximity (**row 3**) from one individual to another. LIKE ranges from 0.99 (black) to 0.01 (white). Grooming ranges from 9.1 (black) to 0 (white). Proximity ranges from 0.58 (black) to 0 (white). The figures show runs using the alternative dynamics and an intermediate increase speed. These simulation runs correspond to the bottom two images in Fig 3B and Fig 3E. See for the comparison between dyadic grooming, proximity and LIKE-values with the original dynamics: [42, Fig 2].

Reference 42. Evers E, De Vries H, Spruijt BM, Sterck EHM (2015) Emotional bookkeeping and high partner selectivity are necessary for the emergence of partner-specific reciprocal affiliation in an agent-based model of primate groups. PLoS ONE 10:e0118921 DOI 10.1371/journal.pone.0118921.

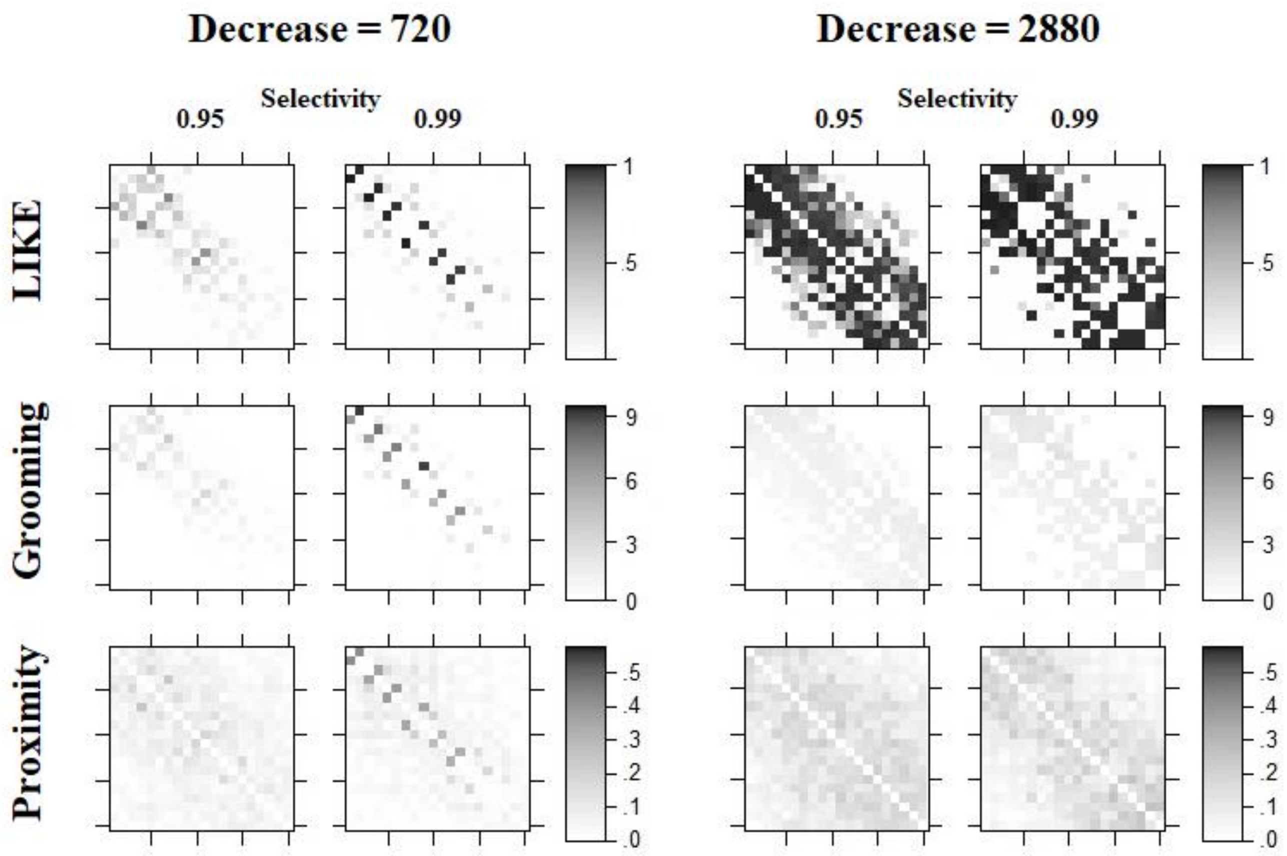

Decrease = 720
Decrease = 2880
Selectivity
0.95
0.99
Selectivity
0.95
0.99
LIKE
Grooming
Proximity
1
.5
9
6
3
0
.5
.4
.3
.2
.1
.0
