# Supplementary material for: Emotional bookkeeping and differentiated affiliative relationships: Exploring the role of dynamics and speed in updating relationship quality in the EMO-model
Source: PLoS One. 2021 Apr 2;16(4):e0249519. doi: 10.1371/journal.pone.0249519 (PMC8018660; doi:10.1371/journal.pone.0249519)
Supplement: S6 Fig — On the y-axis individuals are ordered from low ranking (top row) to high ranking (bottom row). On the x-axis individuals are ordered from low ranking (left) to high ranking (right). Each square represents grooming from one individual to another. Figures a, b and c show the original dynamics with a fast, intermediate and slow increase speed respectively. Figures d, e and f show the alternative dynamics with a fast, intermediate and slow increase speed respectively. Grooming ranges a: From 2.6 (black) to 0 (white); b: 3.4 to 0; c: 5.1 to 0; d: 2.6 to 0; e: 9.1 to 0; f: 11.1 to 0. (PDF) [file pone.0249519.s006.pdf]

Emotional bookkeeping and differentiated affiliative relationships: exploring the role of dynamics and speed in updating relationship quality in the EMO-model

Tonko W Zijlstra, Han de Vries & Elisabeth HM Sterck

Supporting information S7: Dyadic grooming rates

Fig S7: Dyadic grooming rates averaged over the "second year" of the recording period for four different levels of selectivity (LPS) and six different decrease speeds (LHW). On the y-axis individuals are ordered from low ranking (top row) to high ranking (bottom row). On the x-axis individuals are ordered from low ranking (left) to high ranking (right). Each square represents grooming from one individual to another. Figures **a**, **b** and **c** show the original dynamics with a fast, intermediate and slow increase speed respectively. Figures **d**, **e** and **f** show the alternative dynamics with a fast, intermediate and slow increase speed respectively. Grooming ranges **a**: from 2.6 (black) to 0 (white); **b**: 3.4 to 0; **c**: 5.1 to 0; **d**: 2.6 to 0; **e**: 9.1 to 0; **f**: 11.1 to 0.

Dyadic grooming rates; Original dynamics; Fast increase speed

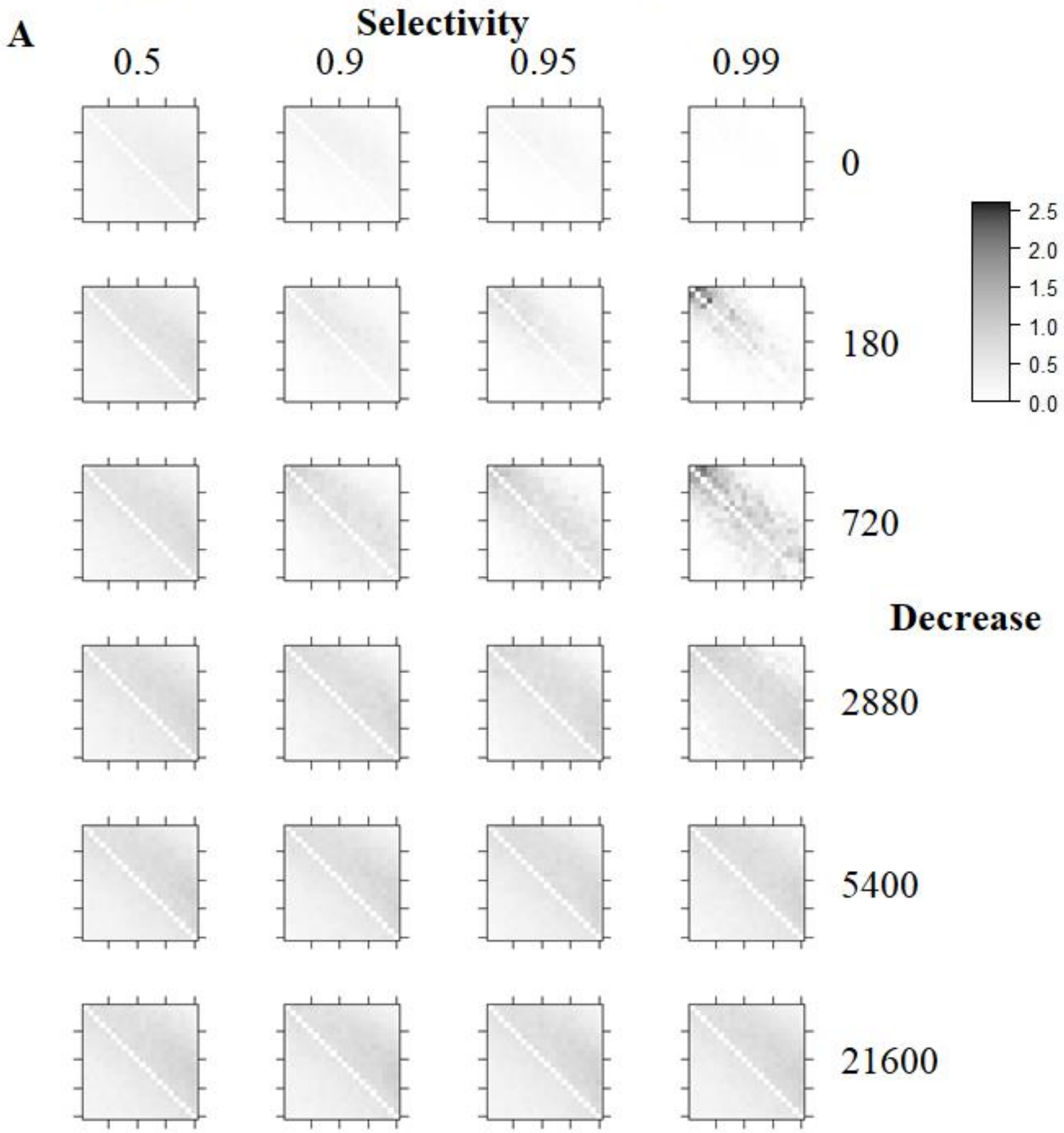

Dyadic grooming rates; Original dynamics; Intermediate increase speed

B

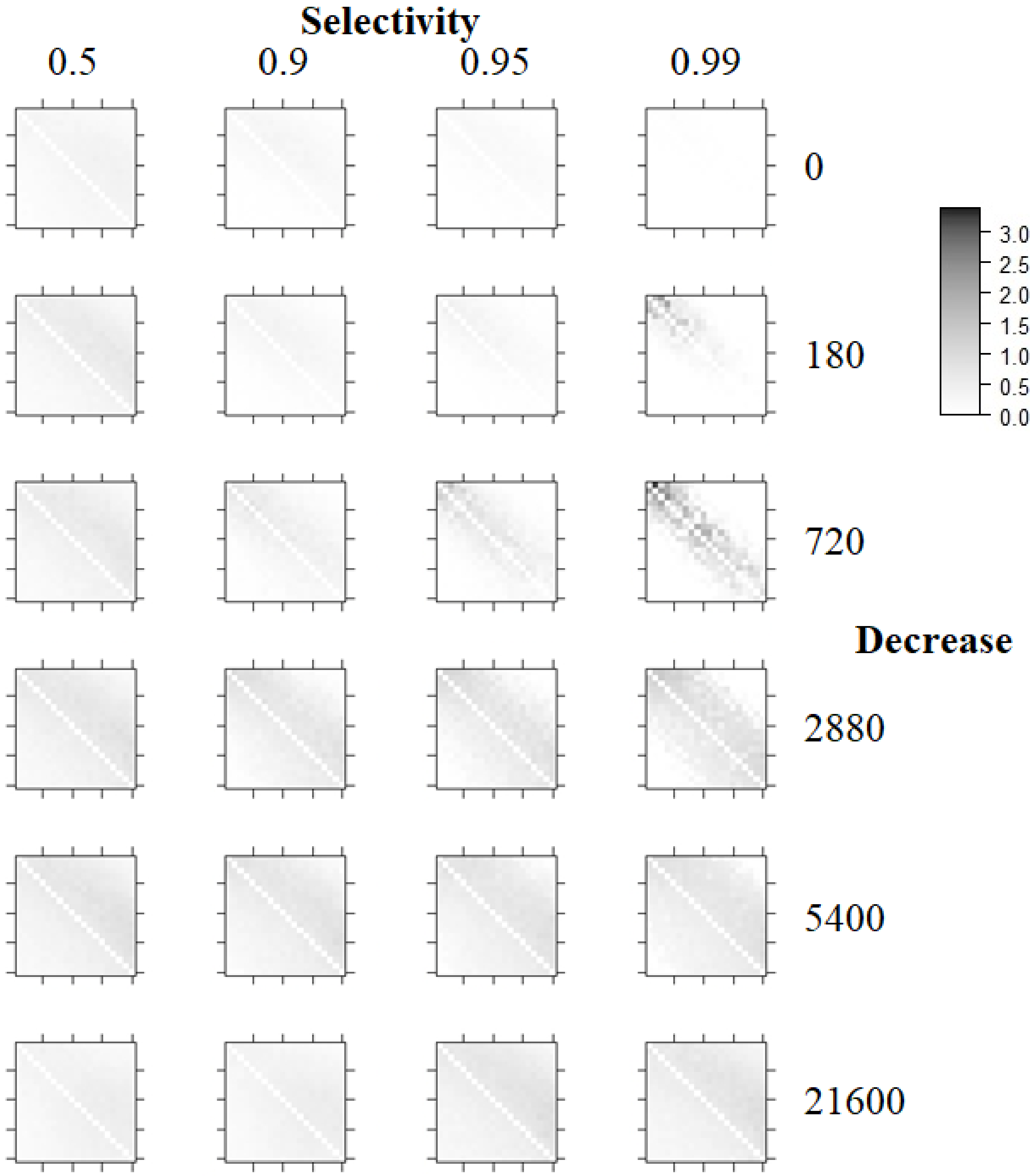

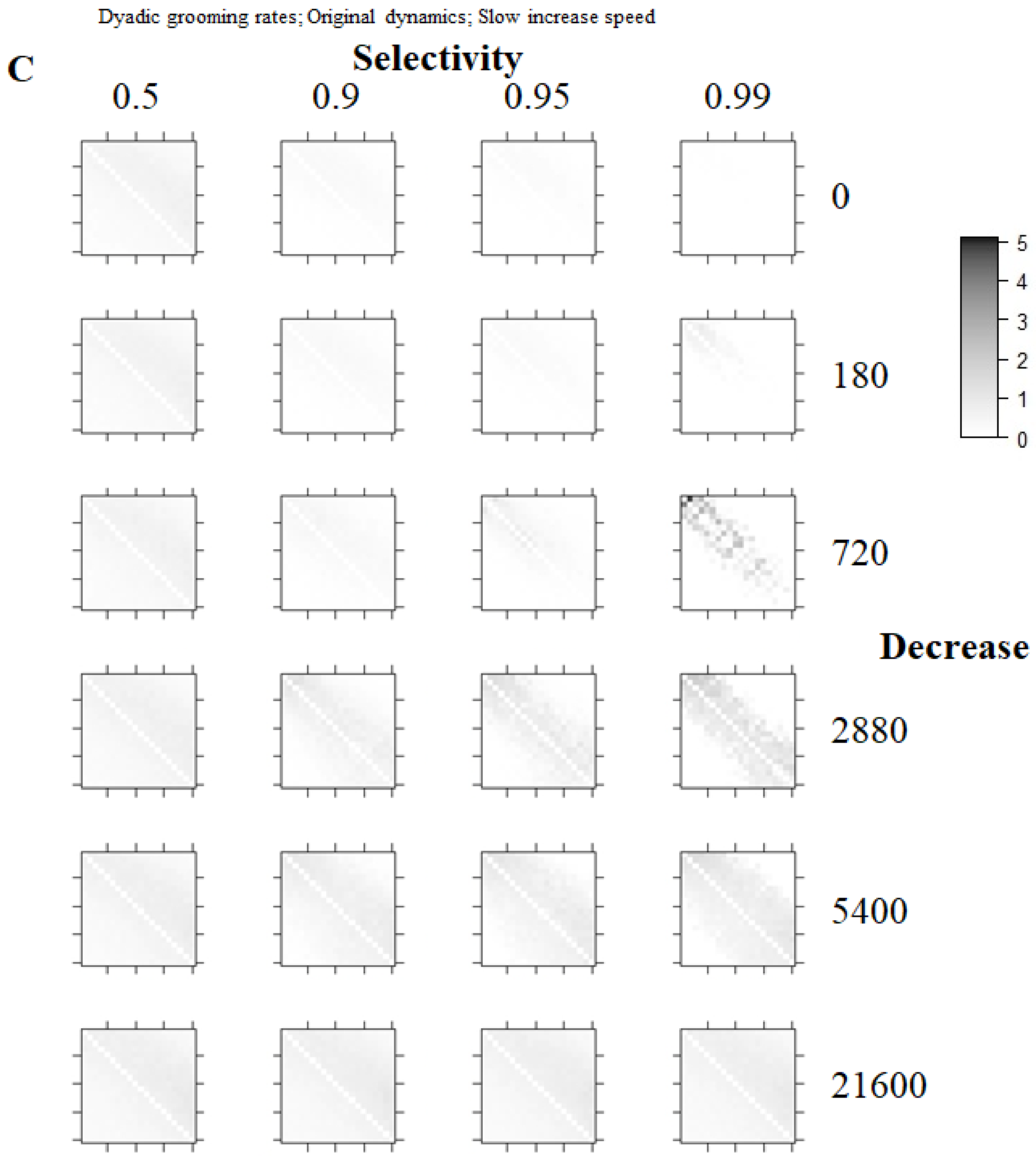
Dyadic grooming rates; Original dynamics; Slow increase speed
C
Selectivity
0.5
0.9
0.95
0.99
0
180
720
Decrease
2880
5400
21600
5
4
3
2
1
0

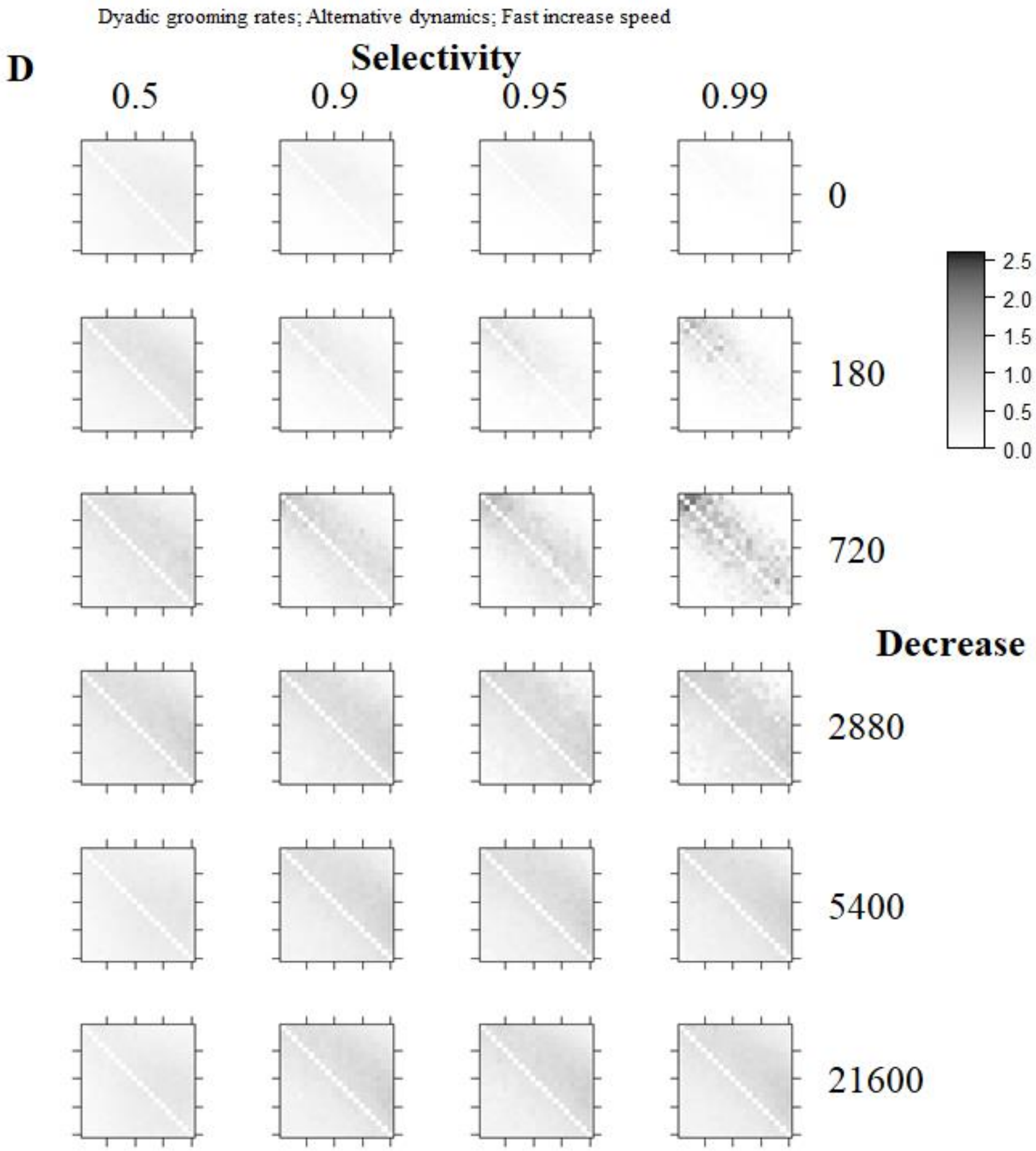
Dyadic grooming rates; Alternative dynamics; Fast increase speed
D
Selectivity
0.5
0.9
0.95
0.99
0
180
720
Decrease
2880
5400
21600
2.5
2.0
1.5
1.0
0.5
0.0

Dyadic grooming rates; Alternative dynamics; Intermediate increase speed

E

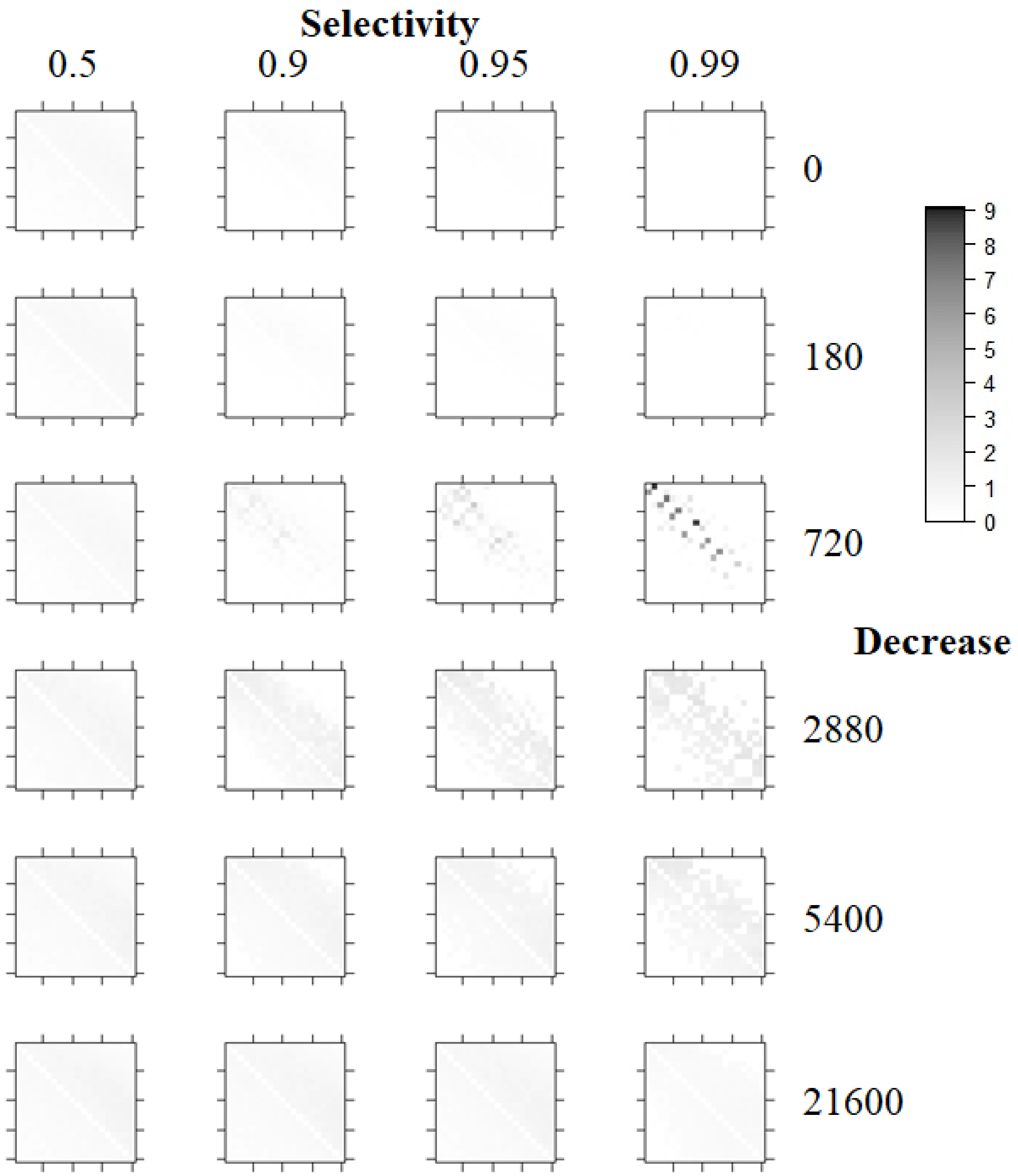

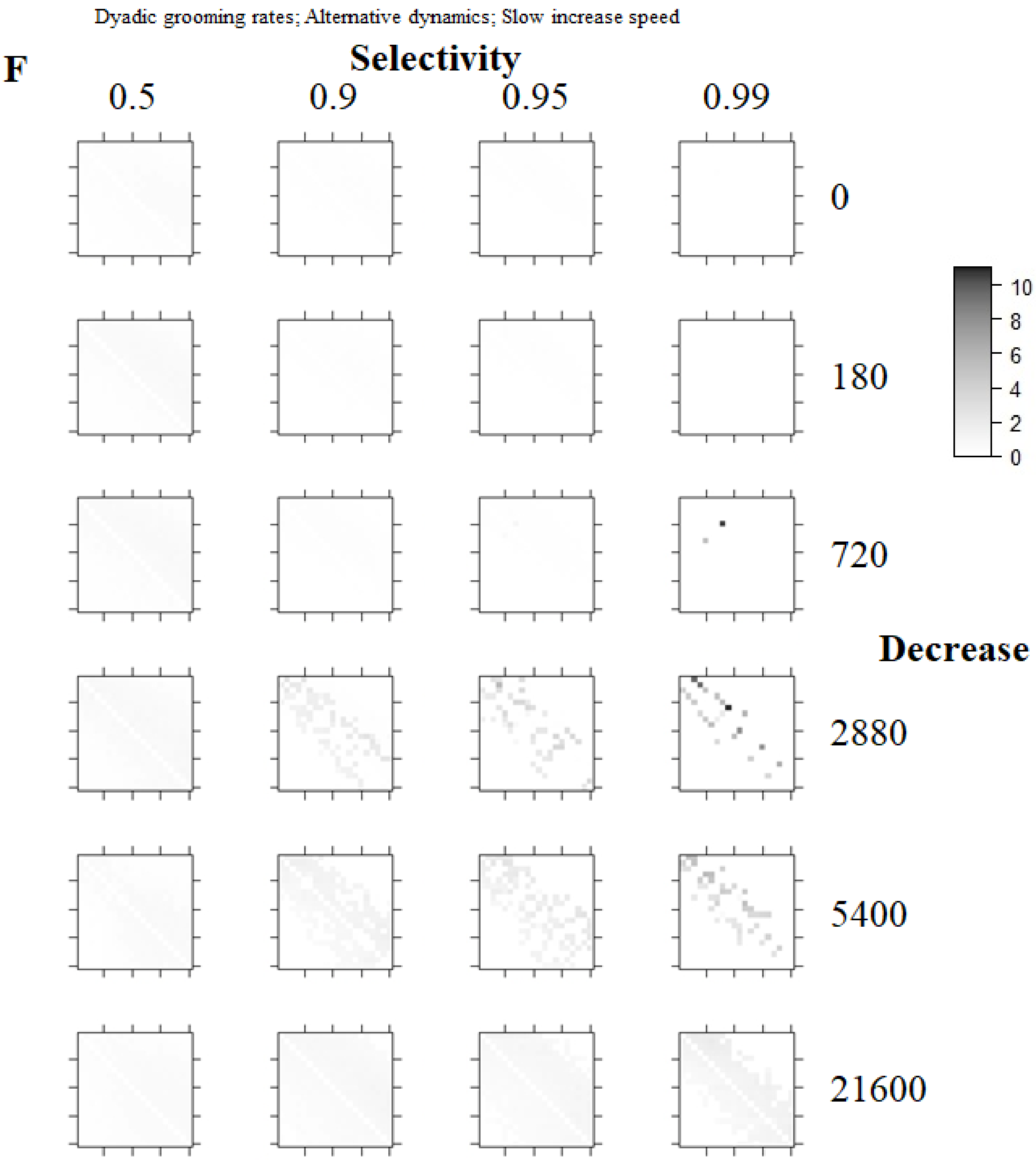
Dyadic grooming rates; Alternative dynamics; Slow increase speed
F
Selectivity
0.5
0.9
0.95
0.99
0
180
720
2880
5400
21600
Decrease
10
8
6
4
2
0
