# Supplementary material for: Emotional bookkeeping and differentiated affiliative relationships: Exploring the role of dynamics and speed in updating relationship quality in the EMO-model
Source: PLoS One. 2021 Apr 2;16(4):e0249519. doi: 10.1371/journal.pone.0249519 (PMC8018660; doi:10.1371/journal.pone.0249519)
Supplement: S7 Fig — On the y-axis individuals are ordered from low ranking (top row) to high ranking (bottom row). On the x-axis individuals are ordered from low ranking (left) to high ranking (right). Each square represents proximity from one individual to another. Figures a, b and c show the original dynamics with a fast, intermediate and slow increase speed respectively. Figures d, e and f show the alternative dynamics with a fast, intermediate and slow increase speed respectively. Proximity ranges from 0.4 (black) to 0 (white) in figures a, b and c: And from 0.58 to 0 in figures d, e and f. (PDF) [file pone.0249519.s007.pdf]

Emotional bookkeeping and differentiated affiliative relationships: exploring the role of dynamics and speed in updating relationship quality in the EMO-model

Tonko W Zijlstra, Han de Vries & Elisabeth HM Sterck

Supporting information S8: Dyadic proximity rates

Fig S8: Dyadic proximity rates averaged over the "second year" of the recording period for four different levels of selectivity (LPS) and six different decrease speeds (LHW). On the y-axis individuals are ordered from low ranking (top row) to high ranking (bottom row). On the x-axis individuals are ordered from low ranking (left) to high ranking (right). Each square represents proximity from one individual to another. Figures **a**, **b** and **c** show the original dynamics with a fast, intermediate and slow increase speed respectively. Figures **d**, **e** and **f** show the alternative dynamics with a fast, intermediate and slow increase speed respectively. Proximity ranges from 0.4 (black) to 0 (white) in figures **a**, **b** and **c**: and from 0.58 to 0 in figures **d**, **e** and **f**.

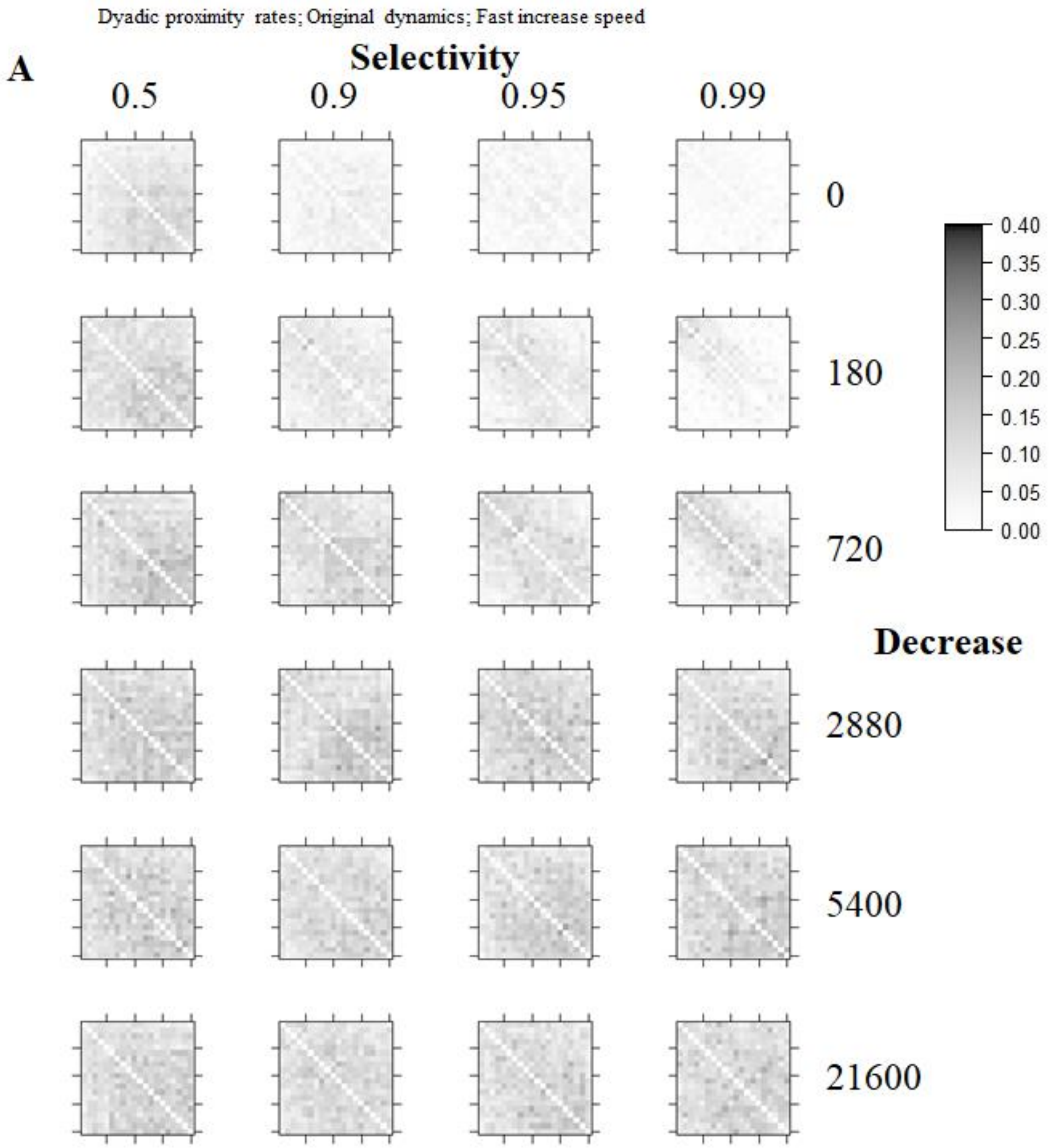
Dyadic proximity rates; Original dynamics; Fast increase speed
A
Selectivity
0.5
0.9
0.95
0.99
0
180
720
2880
5400
21600
Decrease
0.40
0.35
0.30
0.25
0.20
0.15
0.10
0.05
0.00

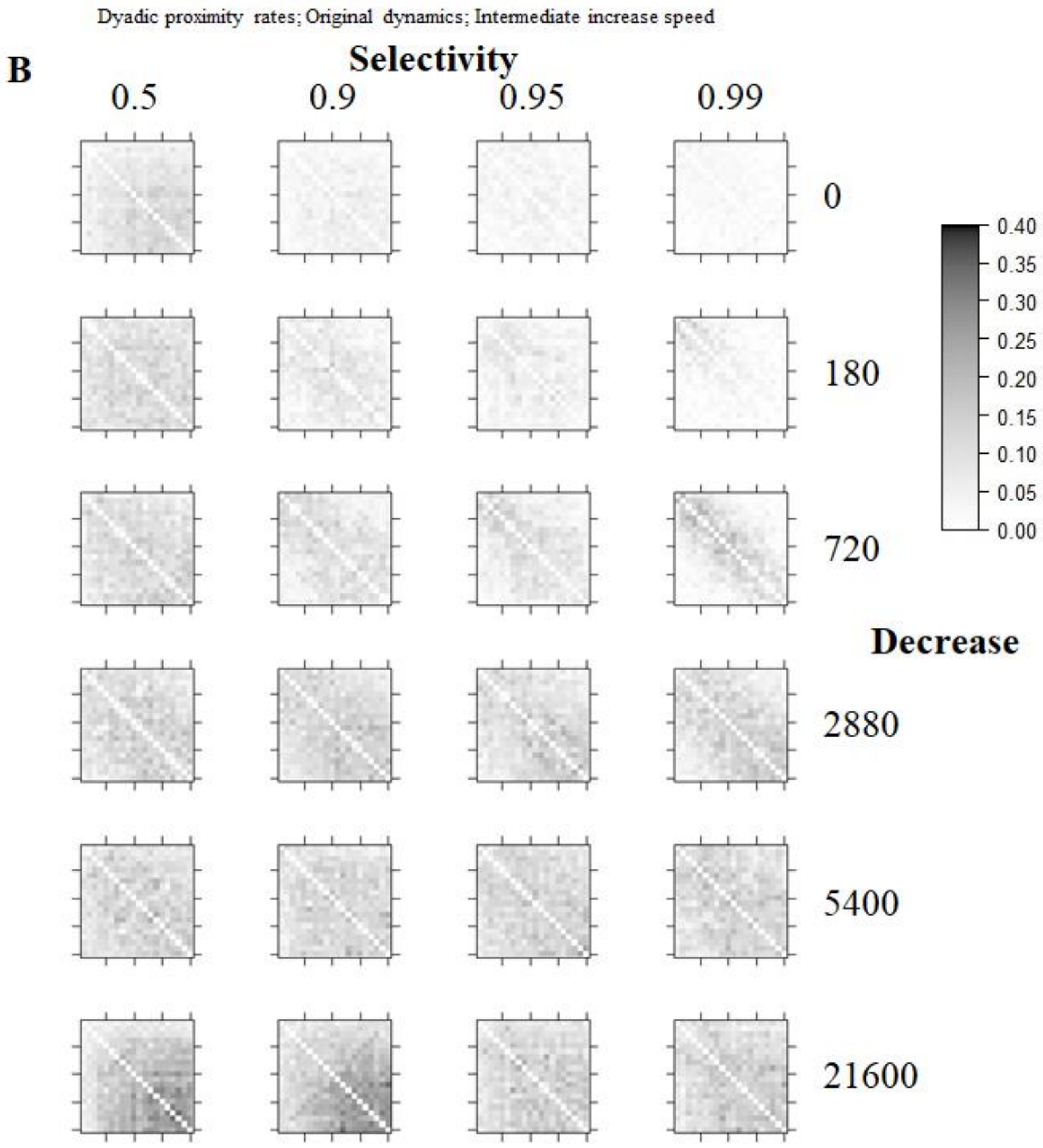

Dyadic proximity rates; Original dynamics; Intermediate increase speed
B
Selectivity
0.5
0.9
0.95
0.99
0
180
720
2880
5400
21600
Decrease
0.40
0.35
0.30
0.25
0.20
0.15
0.10
0.05
0.00

Dyadic proximity rates; Original dynamics; Slow increase speed

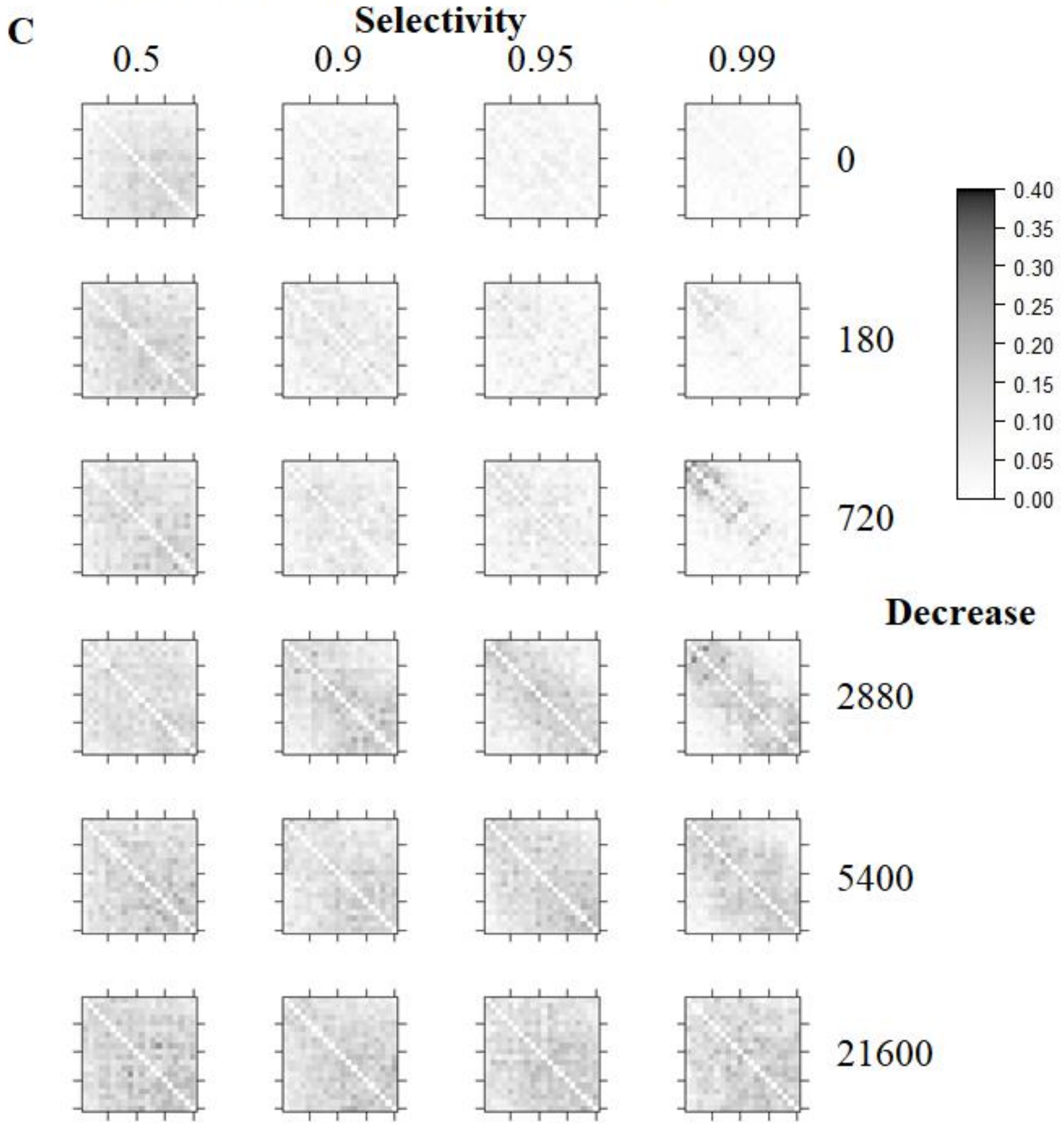

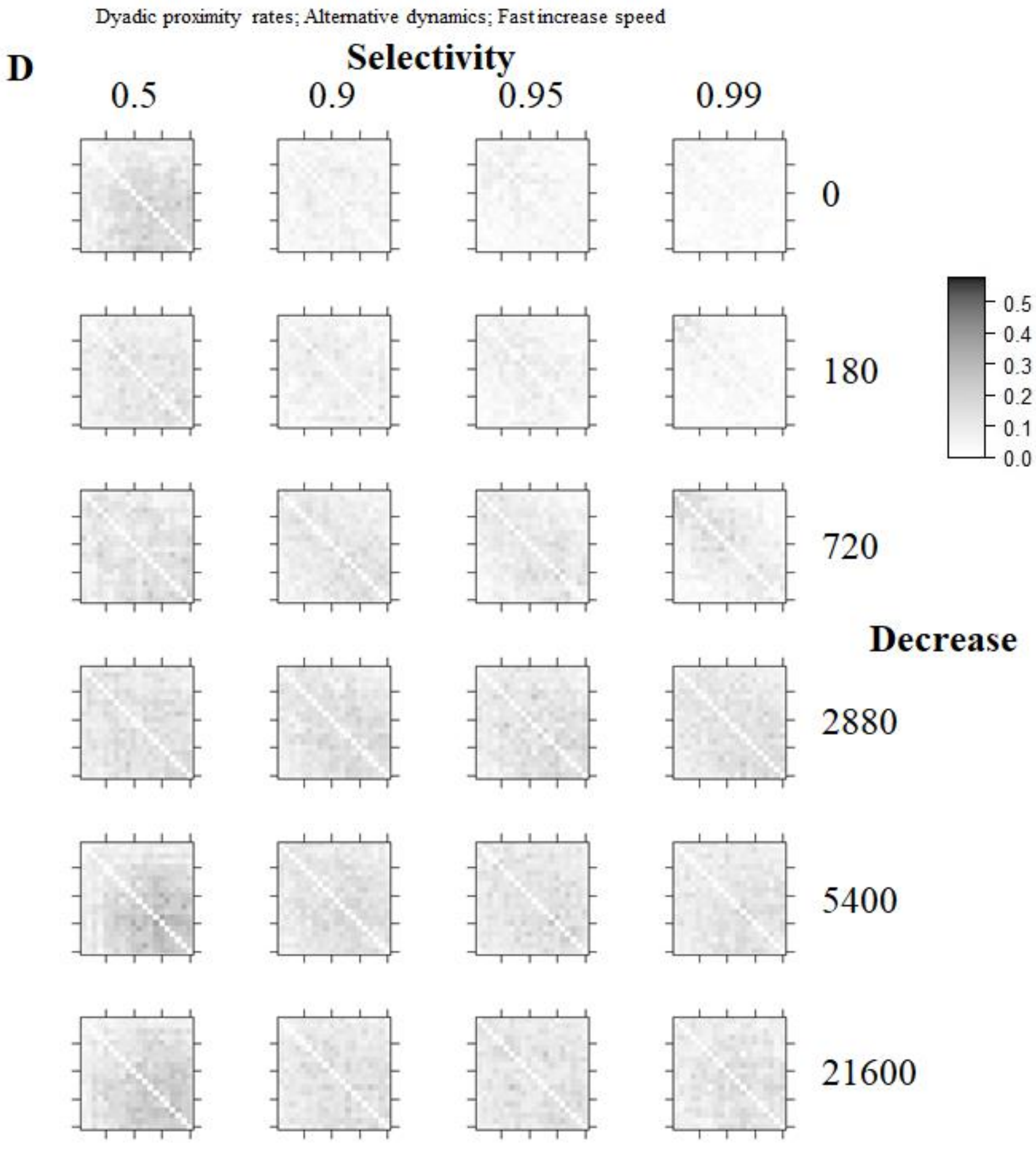
Dyadic proximity rates; Alternative dynamics; Fast increase speed
D
Selectivity
0.5
0.9
0.95
0.99
0
180
720
Decrease
2880
5400
21600
0.5
0.4
0.3
0.2
0.1
0.0

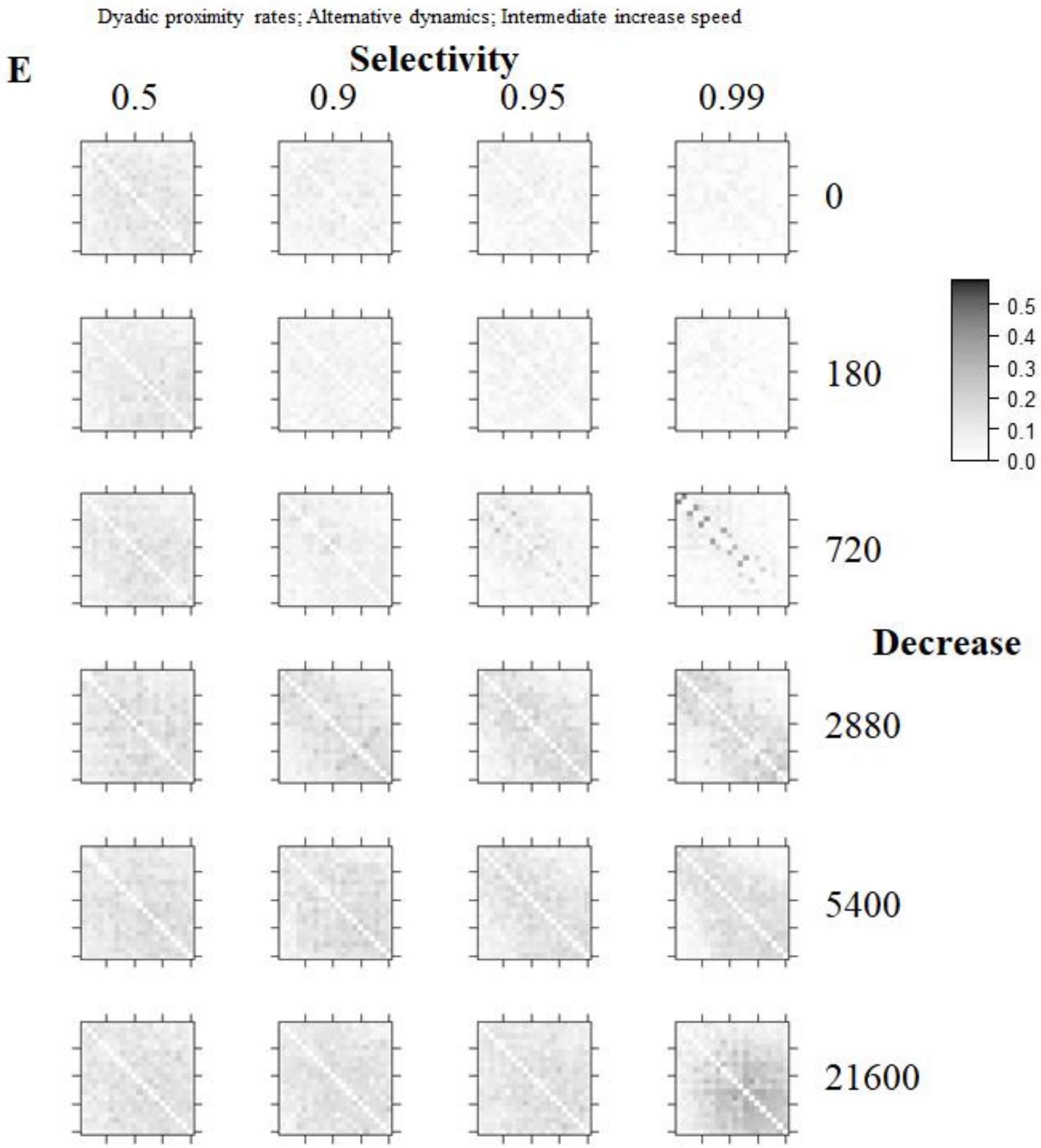
Dyadic proximity rates; Alternative dynamics; Intermediate increase speed
E
Selectivity
0.5
0.9
0.95
0.99
0
180
720
Decrease
2880
5400
21600
0.5
0.4
0.3
0.2
0.1
0.0

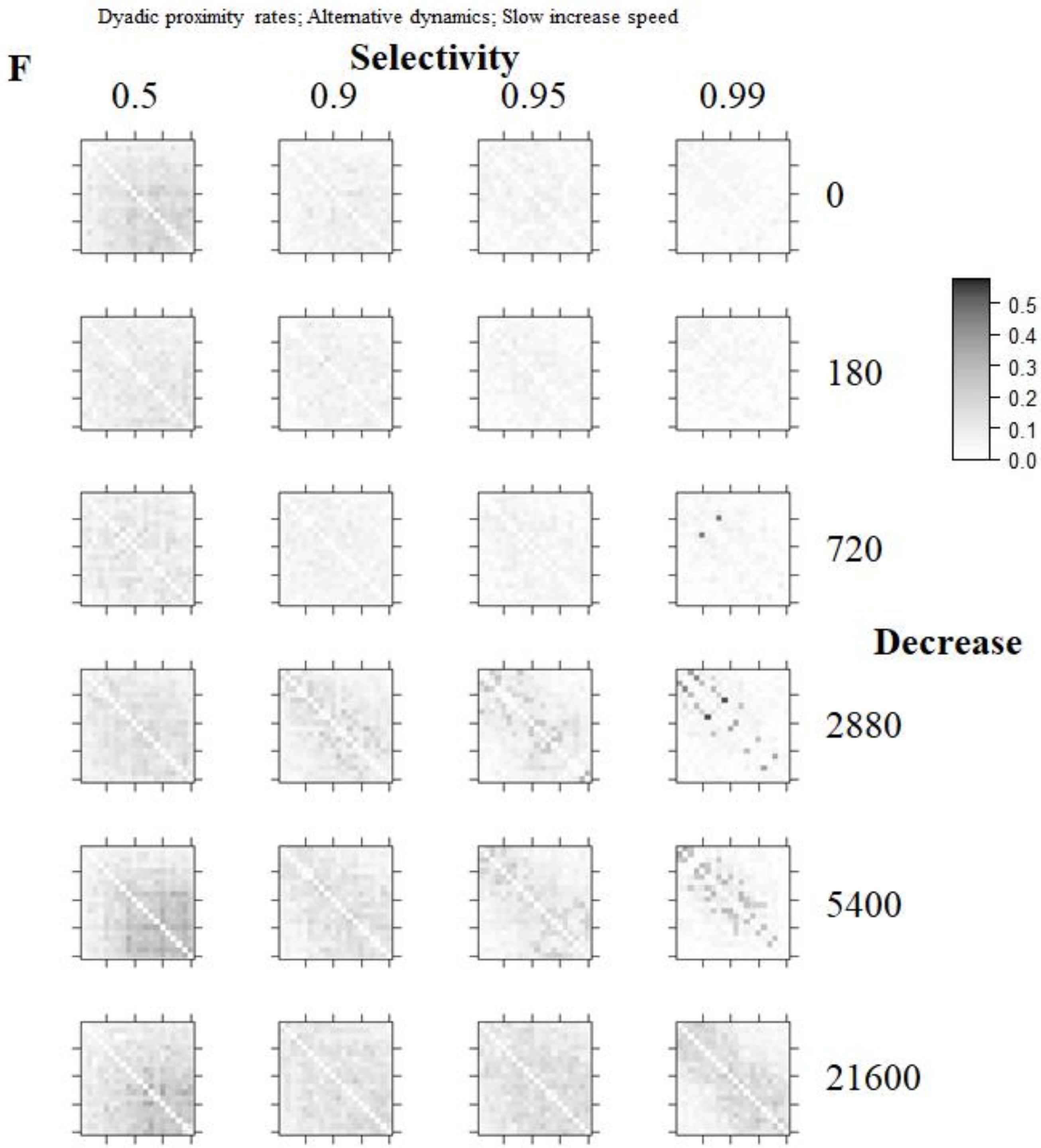
Dyadic proximity rates; Alternative dynamics; Slow increase speed
F
Selectivity
0.5
0.9
0.95
0.99
0
180
720
Decrease
2880
5400
21600
0.5
0.4
0.3
0.2
0.1
0.0
