# Supplementary material for: Emotional bookkeeping and differentiated affiliative relationships: Exploring the role of dynamics and speed in updating relationship quality in the EMO-model
Source: PLoS One. 2021 Apr 2;16(4):e0249519. doi: 10.1371/journal.pone.0249519 (PMC8018660; doi:10.1371/journal.pone.0249519)
Supplement: S8 Fig — Each bar represents all 380 dyadic relationships in a single simulation run. (PDF) [file pone.0249519.s008.pdf]

Emotional bookkeeping and differentiated affiliative relationships: exploring the role of dynamics and speed in updating relationship quality in the EMO-model

Tonko W Zijlstra, Han de Vries & Elisabeth HM Sterck

Supporting information S9: Impact of combined dynamics on differentiation

Fig S9: The number of relationships categorised as high, intermediate and low quality for the original dynamics, dynamics 3, dynamics 4 and the alternative dynamics for two increase speeds (fast and slow), intermediate decrease speed (LHW=2880) and very high partner selectivity (LPS=0.99). Each bar represents all 380 dyadic relationships in a single simulation run.

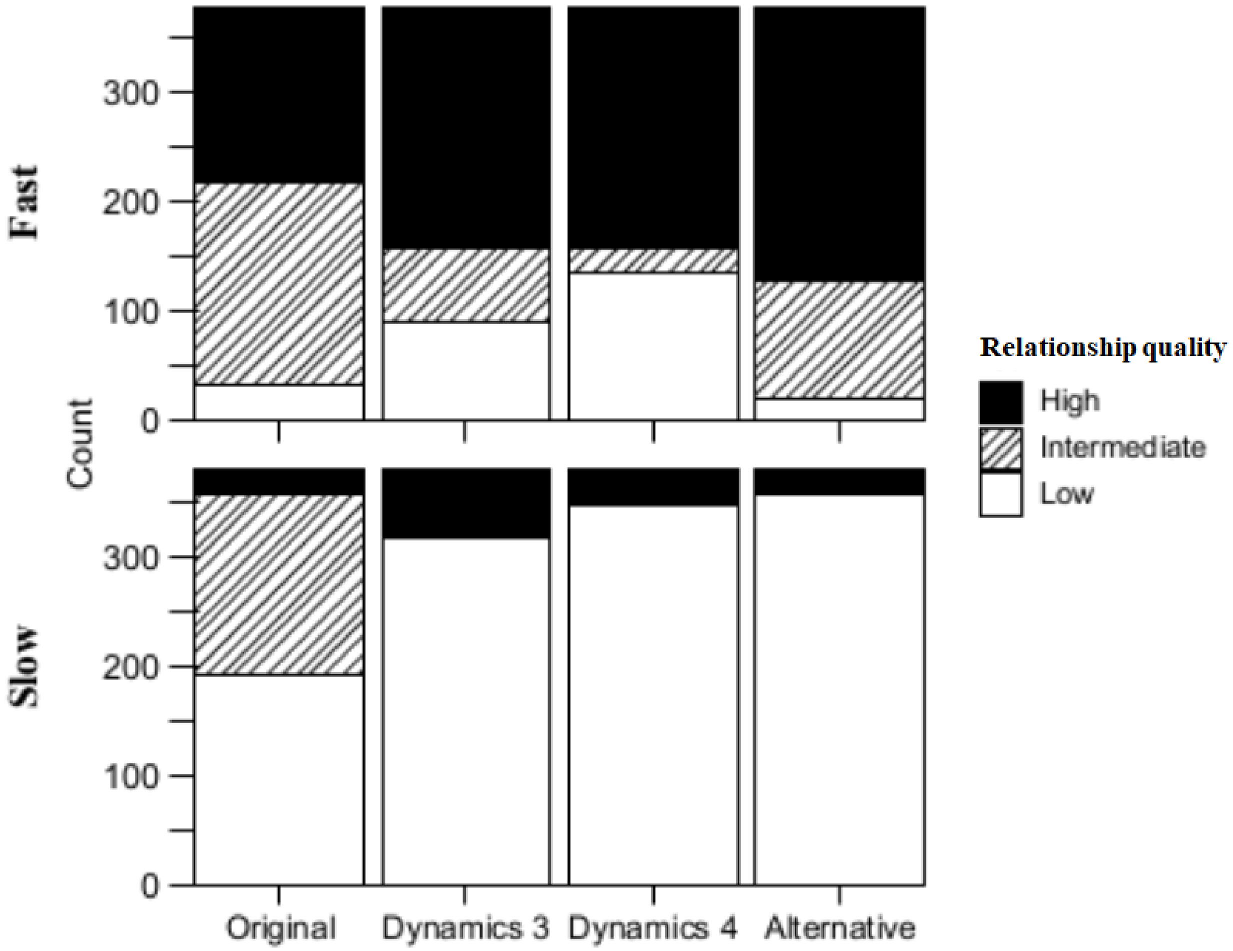
Fast
Slow
Count
300
200
100
0
Original
Dynamics 3
Dynamics 4
Alternative
Relationship quality
High
Intermediate
Low
