# Supplementary material for: Emotional bookkeeping and differentiated affiliative relationships: Exploring the role of dynamics and speed in updating relationship quality in the EMO-model
Source: PLoS One. 2021 Apr 2;16(4):e0249519. doi: 10.1371/journal.pone.0249519 (PMC8018660; doi:10.1371/journal.pone.0249519)
Supplement: S9 Fig — Each column shows dyadic LIKE values at a different point in time, from the LIKE distribution at the start of the recording period (t = 0) to the LIKE distribution at the end of the recording period (t = 2). An R2 value was calculated between adjacent years and the 4 resulting R2 values were averaged. The average R2 is shown to the right of the 5 figures in each row. On the y-axis individuals are ordered from low ranking (top row) to high ranking (bottom row). On the x-axis individuals are ordered from low ranking (left) to high ranking (right). Each square represents LIKE from one individual to another. LIKE ranges from 0.99 (black) to 0.01 (white). Row 1 corresponds to the bottom row in Fig 5C and to the bottom right image in Fig 3F. Row 2 corresponds to the bottom left image in Fig 3F. (PDF) [file pone.0249519.s009.pdf]

Emotional bookkeeping and differentiated affiliative relationships: exploring the role of dynamics and speed in updating relationship quality in the EMO-model

Tonko W Zijlstra, Han de Vries & Elisabeth HM Sterck

Supporting information S10: Stability of LIKE compared between different levels of partner selectivity

Fig S10: Stability of LIKE values over time for the alternative dynamics, intermediate decrease speed (LHW=2880) and four different levels of partner selectivity (LPS). Each column shows dyadic LIKE values at a different point in time, from the LIKE distribution at the start of the recording period (t=0) to the LIKE distribution at the end of the recording period (t=2). An R^2 value was calculated between adjacent years and the 4 resulting R^2 values were averaged. The average R^2 is shown to the right of the 5 figures in each row. On the y-axis individuals are ordered from low ranking (top row) to high ranking (bottom row). On the x-axis individuals are ordered from low ranking (left) to high ranking (right). Each square represents LIKE from one individual to another. LIKE ranges from 0.99 (black) to 0.01 (white). Row 1 corresponds to the bottom row in Fig 5C in the main text and to the bottom right image in Fig 3F in the main text. Row 2 corresponds to the bottom left image in Fig 3F in the main text.

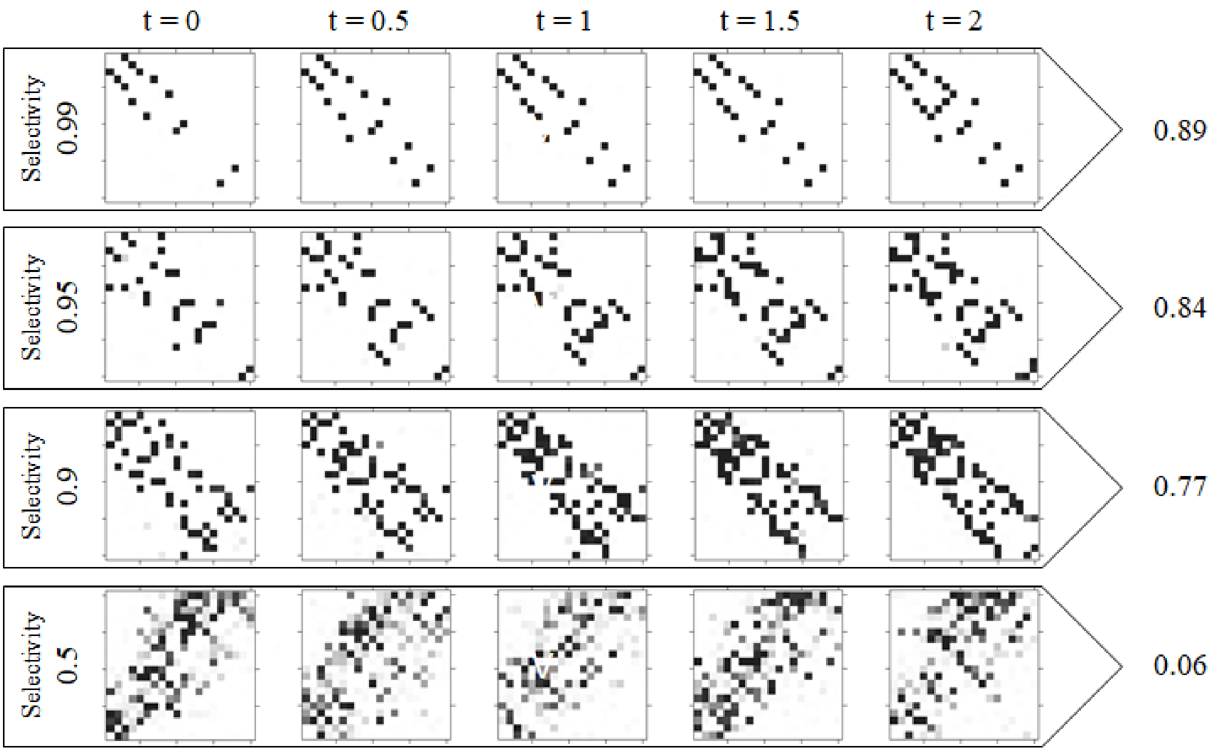

t = 0
t = 0.5
t = 1
t = 1.5
t = 2
Selectivity 0.99
0.89
Selectivity 0.95
0.84
Selectivity 0.9
0.77
Selectivity 0.5
0.06
