# Supplementary material for: Emotional bookkeeping and differentiated affiliative relationships: Exploring the role of dynamics and speed in updating relationship quality in the EMO-model
Source: PLoS One. 2021 Apr 2;16(4):e0249519. doi: 10.1371/journal.pone.0249519 (PMC8018660; doi:10.1371/journal.pone.0249519)
Supplement: S1 Table — (PDF) [file pone.0249519.s010.pdf]

Emotional bookkeeping and differentiated affiliative relationships: exploring the role of dynamics and speed in updating relationship quality in the EMO-model

Tonko W Zijlstra, Han de Vries & Elisabeth HM Sterck

Supporting information S1: Values used in the logistical formula

S1 Table: Levels of steepness used for the different increase and decrease speeds of LIKE

**Table S1.1**: 'k' used for the different decrease speeds of LIKE	
LHW (Decrease speed)	Logistic 'k'
0	0.18380
180	0.02553
720	0.00638
2880	0.00160
5400	0.00085
21600	0.00021

**Table S1.2**: 'k' and 'a' used for the different increase speeds of LIKE		
LINC (Increase speed)	Logistic 'k'	Linear 'a'
Fast	0.91902	0.1
intermediate	0.30634	0.03333
Slow	0.15317	0.01667
